# DNA-enabled rational design of fluorescence-Raman bimodal nanoprobes for cancer imaging and therapy

**DOI:** 10.1038/s41467-019-09173-2

**Published:** 2019-04-26

**Authors:** Suchetan Pal, Angana Ray, Chrysafis Andreou, Yadong Zhou, Tatini Rakshit, Marek Wlodarczyk, Masatomo Maeda, Ricardo Toledo-Crow, Naxhije Berisha, Jiang Yang, Hsiao-Ting Hsu, Anton Oseledchyk, Jagannath Mondal, Shengli Zou, Moritz F. Kircher

**Affiliations:** 1 Center for Molecular Imaging and Nanotechnology (CMINT), Memorial Sloan Kettering Cancer Center, New York, NY 10065 USA; 2 Department of Radiology, Memorial Sloan Kettering Cancer Center, New York, NY 10065 USA; 3 Tata Institute of Fundamental Research, Hyderabad, Telangana 500107 India; 4 Department of Chemistry, University of Central Florida, Orlando, FL 32816 USA; 5 Department of Bioengineering, New York University, New York, NY 10010 USA; 6 Ph.D. Program in Chemistry, The Graduate Center of the City University of New York, New York, NY 10016 USA; 7 Advanced Science Research Center, City University of New York, New York, NY 10031 USA; 8 Molecular Pharmacology Program, Sloan Kettering Institute, New York, NY 10065 USA; 9 Department of Radiology, Weill Cornell Medical College, New York, NY 10021 USA; 10 Department of Imaging, Dana-Farber Cancer Institute, Boston, MA 02215 USA; 11 Present Address: Department of Chemistry, Indian Institute of Technology Bhilai, Raipur, Chhattisgarh 492015 India

**Keywords:** Nanoparticles, Cancer imaging, Cancer therapy

## Abstract

Recently, surface-enhanced Raman scattering nanoprobes have shown tremendous potential in oncological imaging owing to the high sensitivity and specificity of their fingerprint-like spectra. As current Raman scanners rely on a slow, point-by-point spectrum acquisition, there is an unmet need for faster imaging to cover a clinically relevant area in real-time. Herein, we report the rational design and optimization of fluorescence-Raman bimodal nanoparticles (FRNPs) that synergistically combine the specificity of Raman spectroscopy with the versatility and speed of fluorescence imaging. DNA-enabled molecular engineering allows the rational design of FRNPs with a detection limit as low as 5 × 10^−15^ M. FRNPs selectively accumulate in tumor tissue mouse cancer models and enable real-time fluorescence imaging for tumor detection, resection, and subsequent Raman-based verification of clean margins. Furthermore, FRNPs enable highly efficient image-guided photothermal ablation of tumors, widening the scope of the NPs into the therapeutic realm.

## Abstract (web summary)

Currently available Raman scanners are limited in speed to acquire images of clinically relevant sizes in cancer imaging. Here, the authors developed a DNA based design principle for Raman-Fluorescence bimodal nanoparticles and demonstrate real-time, high precision image-guided tumor resections and photothermal ablation of cancer.

## Introduction

In the last decade, multifunctional and multimodal nanoparticles (NPs) have emerged as promising tools for oncological imaging and therapy^1,2^. One of the advantages of NP-based theranostics is the synergistic amalgamation of multiple complementary imaging modalities and therapeutic capabilities in a single nanoformulation. Due to their nanoscale dimensions, NPs selectively accumulate in tumor tissue, can enable diagnosis, and aid in the precise surgical removal of malignant tissue and subsequent mitigation of the residual malignancies^3^. Amongst all imaging modalities, optical imaging provides unique advantages over other non-optical modalities (e.g., PET, SPECT, CT, MRI), such as no harmful radiation exposure, high sensitivity, and better spatiotemporal resolution^4^. For in vivo imaging applications, optical imaging probes in the near-infrared (NIR) window (650–900 nm) are preferred because this range offers enhanced tissue penetration depth due to reduced light absorption by hemoglobin and water^5^. Therefore, a plethora of NIR-based imaging probes are being designed and tested in preclinical and clinical settings for diagnosis and intraoperative cancer imaging^6–8^. While NIR fluorescence is one of the most popular imaging modalities, it suffers from drawbacks such as tissue autofluorescence and photobleaching, which poses potential limitation of its use in the clinical setting.

In recent years, Raman imaging (both intrinsic and SERS NP contrast agent based) is being recognized as a viable alternative to NIR fluorescence imaging^9–11^. Particularly, SERS NP-based imaging has demonstrated an unparalleled accuracy of tumor delineation due to the highly specific, “fingerprint”-like, biorthogonal Raman spectral signatures. Unlike most of the fluorescence-based NPs, SERS NPs do not suffer from tissue autofluorescence or photobleaching. A recent preclinical study assessing the toxicity of SERS NPs in mice found no significant adverse effects apart from a mild inflammatory response^12^. Due to these advantages, SERS NP-based imaging methods are gaining substantial momentum and are being successfully utilized to delineate exact tumor margins in various cancers, micrometastases, and even precancerous lesions with very high precision in pre-clinical settings^13–17^. More encouragingly, SERS NPs surface-displaying targeting moieties such as antibodies, aptamers, or targeting peptides (e.g., RGD) were shown to delineate tumors more effectively via active targeting in instances where passive targeting is relatively weak^18–23^. The increased precision of imaging the true microscopic tumor margins could reduce unnecessary resection of surrounding healthy tissue and also facilitate surgeries that are presently not feasible because of the proximity of crucial structures such as nerves or blood vessels. Further, SERS NPs might enable minimally invasive or robotically assisted surgical approaches in situations where currently open surgical approaches are preferred. Although SERS NPs offer high sensitivity and specificity in cancer imaging, the technique currently requires a time-intensive point-by-point acquisition of Raman spectra, precluding the real-time image acquisition desired for clinical applications. Although a recently developed high-speed wide field Raman scanner reports a 10-fold decrease in scan time, acquisition still requires time in the order of several minutes for small animals^24^. Therefore, there exists an unmet need for developing a multimodal NP that combines fast NIR fluorescence-based intraoperative imaging with the highly specific Raman-based cross-validation and clean margin confirmation. Further, additional therapeutic capabilities would enable the NPs to mitigate any residual malignancies after surgery.

Current SERS NP design considerations use chalcogenide-containing and/or positively charged NIR fluorophores as Raman reporters, that attach to negatively charged AuNP surface via covalent or electrostatic interactions, respectively^25^. However, the close proximity to the AuNP surface leads to high fluorescence quenching due to nonradiative energy transfer^26^. To remedy this, additional fluorophores or quantum dots are attached to the nanoparticle to restore fluorescence-based imaging capabilities^27–30^. In this report, we present the DNA-based rational design of an all-in-one, dual-mode fluorescence-Raman NP (referred to as FRNP hereafter) for cancer imaging and therapy. The motivation behind using DNA as a structural component of the FRNP is the recent substantial advancement in the field of DNA-based nanotechnology^31^. This emerging, interdisciplinary research field utilizes DNA as a construction component for materials with emerging properties and is expanding its scope in many advanced technological facets^32,33^. Due to the programmable nature of DNA, it is feasible to tailor the structural and functional properties of plasmonic and photonic nanomaterials for many desired applications^34–36^. Therefore, we explored the possibility of using DNA as a programmable linker between the AuNP and the fluorophore for the optimization of an FRNP design to achieve high sensitivity of both fluorescence and SERS combined with photothermal therapy (PTT) enabled tumor ablation capabilities using in vivo models of cancer.

## Results

### Design considerations for FRNPs

NIR fluorophores (typically used as Raman reporters) emit both fluorescence and Raman light when excited by a NIR light source. However, the fluorescence emission is significantly brighter than the intrinsic Raman intensity. Therefore, peaks of Raman emission are overshadowed by the fluorescence emission. Fascinatingly, the close proximity of a fluorophore to a plasmonic NP surface induces a substantial enhancement of the intrinsic Raman intensity (by factors up to 10^15^) giving rise to surface-enhanced Raman scattering (SERS)^37,38^. For maximum SERS enhancement, three considerations are taken into account: (i) maximization of Raman reporters onto the NP surface; (ii) minimization of the reporter’s distance to the NP surface and therefore maximization of the electric field enhancement experienced by the molecule; and (iii) implementation of resonance Raman scattering (i.e., when the excitation laser wavelength matches the absorbance maximum of the electronic transition of the molecule)^39^. Typically, the proximity of the Raman reporter to the AuNP surface quenches any fluorescence the molecule would emit, via non-radiative energy transfer to the metal^40^. Therefore, an even higher electric field enhancement is required to compensate for the energy lost due to the non-radiative energy transfer to enhance the fluorescence intensity^41^.

**Fig. 1:**
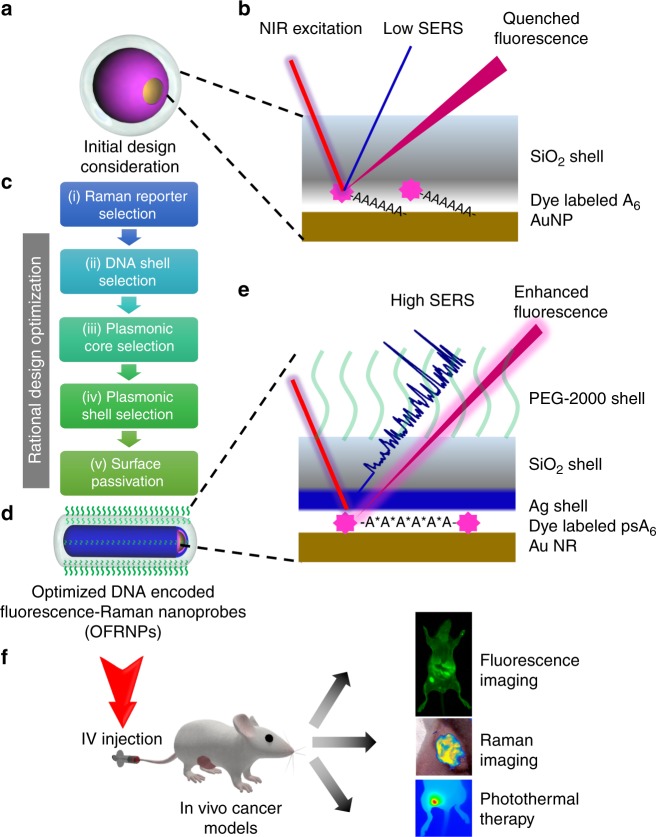
Schematic design principle of FRNPs. **a** We conducted rational design optimization steps (**c**) to transform the initial design consideration into the optimized (**d**) design. **b**, **e** are the blown-up views of the surface for the initial design and optimized design (**d**), respectively. **f** The optimized FRNPs are used for dual-mode cancer imaging and photothermal therapy

In this current work, we formulated a DNA-based strategy that combines ultra-sensitive SERS and fluorescence modalities using an all-in-one fluorophore in an optimized design of FRNP. To date, SERS NPs designed for biomedical applications typically consist of three major components: (i) an Au-based core, (ii) a layer of fluorophore (Raman reporter) on the AuNP surface, and (iii) a protective layer such as silica, protein, or polymer. Taking inspiration from the existing designs, we initially considered a construct consisting of a 60-nm spherical AuNP core, a fluorophore-labeled 6 nucleotides long oligo-A DNA shell, and a silica shell (Fig. 1a, b). From a large sequence space, we chose an A6 DNA because it was the shortest DNA that could be used to functionalize AuNPs without causing aggregation and had higher affinity to Au surface. However, the initial construct was found to be dim in both Raman and fluorescence signals with a limit of detection of 10 nM. To make the FRNPs brighter, we conducted five layers of design refinements, viz., (i) Raman reporter selection, (ii) DNA-shell selection, (iii) plasmonic core selection, (iv) plasmonic shell selection, (v) surface passivation, all described in the following sections (Fig. 1c). After these optimization steps, both Raman and fluorescence signals were enhanced by several orders of magnitude and the FRNPs could be detected at a low fM regime. The optimized structures consisted of a 40 nm by 10 nm gold nanorod (AuNR) core, with three consecutive layers: Dylight^TM^ 780 labeled phosphorothioate backbone modified A6 ((PS)A6) DNA, a thin silver shell, and a 15–20 nm silica shell (Fig. 1d, e).

**Fig. 2:**
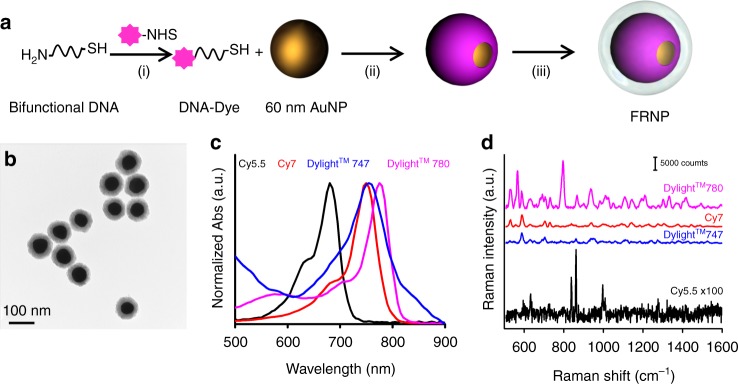
Raman reporter selection. **a** Fabrication of FRNPs. (i) A bifunctional amine-thiol modified DNA is labeled with a NIR fluorophore via straightforward NHS ester chemistry. (ii) Thiol-ends of the fluorophore-labeled DNAs are grafted on to the AuNP surface to create a fluorophore shell. (iii) NPs are silicated using a modified Stöber method. **b** Representative TEM image of the FRNPs. **c** The UV–vis absorption spectra of the 4 different fluorophores selected. **d** The representative baseline-subtracted Raman spectra of 100 fM of the FRNPs synthesized using the 4 fluorophores

In a typical initial FRNP synthesis, an amine-reactive N-hydroxysuccinimide (NHS) group functionalized fluorophore was attached to the amine end of a 5′-thiol and 3′-amine modified bifunctional A6 sequence via straightforward NHS-ester reaction (Fig. 2a). The thiol end of the DNA was attached to a 60 nm AuNP to create a monolayer of NIR fluorophores. Then, a 10–15 nm thick silica layer was formed to enhance the biostability. A representative transmission electron microscope (TEM) image of the non-optimized NPs clearly showed an electron dense 60 nm AuNP core and 20 nm thick SiO_2_ shell with a narrow size distribution (Fig. 2b). We describe the step-by-step design optimization rationale in the following sections.

### Raman reporter selection

First, we sought to select a fluorophore with the maximum Raman cross-section at the excitation wavelength of 785 nm. In reality, a plethora of commercially available NIR fluorophores could be attached to an amine modified DNA via a simple NHS ester chemistry. We chose four NIR fluorophores, Cy5.5 (684 nm excitation maximum, 710 nm emission maximum), Cy7 (750 nm excitation maximum, 775 nm emission maximum), Dylight^TM^ 747 (751 nm excitation maximum, 774 nm emission maximum), and Dylight^TM^ 780 (785 nm excitation maximum, 794 nm emission maximum) for screening (Fig. 2c). We synthesized FRNPs using a bifunctional A6 DNA as shown in Fig. 2a. All the FRNPs expressed finger-print like Raman spectra corresponding to the fluorophore and therefore producing four different flavors of FRNPs (Fig. 2d). We also noted that upon the formation of the silica layer, there was a bathochromic shift of the plasmonic peak and enhanced Raman peak intensities by ~10-fold (Supplementary Fig. 1). We observed that fluorescence baseline subtracted Raman intensities of different flavors of NPs of the same concentration were drastically different. FRNPs derived from Dylight^TM^ 780 labeled A6 DNA demonstrated the highest Raman intensity compared to their other three counterparts (10^3^-fold brighter at the highest Raman peak at 796 cm^−1^ compared to Cy5.5 at the highest Raman peak at 1002 cm^−1^). Therefore, Dylight^TM^ 780 was selected as the lead candidate for the downstream optimization.

### DNA-shell selection

According to the electromagnetic enhancement theory, the SERS enhancement factor decreases very rapidly away from the NP surface. The overall distance dependence should scale with *d*^−12^ for spherical NPs, where *d* is the surface-to-fluorophore distance. Further, for discrete spherical NPs, the average SERS intensity is linearly proportional to the number of fluorophore molecules attached to the NP surface^39^. Therefore, we sought to minimize the fluorophore-surface distance and maximize the fluorophore loading to maximize the Raman signal. It has been determined that adenine (A) has higher affinity to the Au surface compared to other nucleobases (guanine (G), thiamine (T), and cytosine(C))^42^. Therefore, oligo-A DNA molecules tend to bend onto the AuNP surface as predicted by theory and experiments^43,44^. Recently, it has been shown that phosphorothioate (PS) backbone modified poly-A enhanced the DNA loading onto AuNP surface compared to the standard phosphate (PO) backbone^45^.

**Fig. 3:**
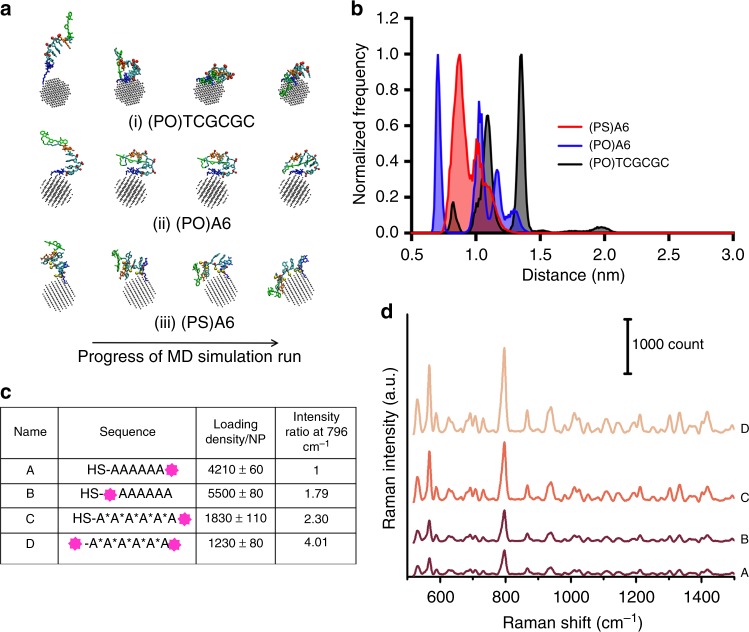
DNA shell selection. **a** Snapshots of (i) HS-(PO)TCGCGC-Cy7, (ii) HS-(PO)A6-Cy7, (iii) HS-(PS)A6-Cy7 DNA folding onto 1.2 nm AuNP surface at different time points of molecular dynamics simulations. Color coding, green: Cy7, orange: 3′ residue, blue: 5′ residue, red balls: O-atoms, yellow balls: S-atoms, gray: Au atoms. **b** The AuNP surface-fluorophore distance distributions of HS-(PO)A6-Cy7 (blue, *d*_average_ = 0.97 nm), HS-(PS)A6-Cy7 (red, *d*_average_ = 0.92 nm), and HS-(PO)TCGCGC-Cy7 (black, *d*_average_ = 1.22 nm) at equilibrium showing very close proximity of the fluorophore to the AuNP surface for first two sequences. **c** Specifications of the 4 different DNA sequences functionalized with Dylight^TM^-780 fluorophores. **d** The baseline-subtracted Raman-spectra of FRNPs (10 fM) derived from the sequences A–D demonstrating that FRNPs derived from sequence D have the highest signal intensity

To verify whether the fluorophore attached to A6 sequences adsorbed at very close proximity to the Au surface, we carried out extensive molecular dynamics (MD) simulations (see SI for details). For the sake of simplicity, we chose an Au-55 core (1.2 nm in diameter) attached to an A6 sequence with a native PO backbone ((PO)A6), a modified PS backbone ((PS)A6), and a control sequence ((PO)TCGCGC). All DNA molecules were flanked by an alkyl thiol at the 5′ end anchored to the AuNP surface and a Cy7 fluorophore (76 Dalton lower molecular weight than Dylight^TM^780) at the 3′ end. We have performed each simulation multiple times independently with different initial seeds, reaching a cumulative of 1 µs long MD-run. The HS-(PS)A6-Cy7 sequence folded onto the AuNP surface starting from perpendicular orientation showing significant affinity of both the sequences to the AuNP surface. However, control DNA sequence (HS-(PO)TCGCGC-Cy7) and HS-(PO)A6-Cy7 demonstrated relatively less bending onto the AuNP surface (Fig. 3a, Supplementary Movies 1–3). Furthermore, the radius of gyration for HS-(PS)A6-Cy7 was higher than HS-(PO)A6-Cy7 and HS-(PO)TCGCGC-Cy7 indicating higher affinity of PS backbone modification DNA sequence to AuNP surface compared to the unmodified sequence (Supplementary Fig. 2). Further, the average Cy7-AuNP surface distance was found to be within 1 nm for both HS-(PO)A6-Cy7 (0.93 nm) and HS-(PS)A6-Cy7 (0.97 nm) DNAs compared to HS-(PO)TCGCGC-Cy7 (1.22 nm) (Fig. 3b). We postulated that a similar folding of the A6 DNA would occur when Dylight^TM^ 780 labeled DNA strands were grafted onto a 60 nm AuNPs because of similarity of interactions.

Results from computational predictions enabled us to select favorable DNA sequences that might be useful for minimizing the surface-to-fluorophore distance. For this optimization step, we designed 4 different Dylight^TM^ 780 modified DNAs (A–D) shown in the table in Fig. 3c. Sequences A and B were based on a PO backbone; A and B were modified with the Dylight^TM^ 780 modification at the nearest and furthermost positions from the thiol modification, respectively. On the other hand, sequences C and D were modified by PS backbone with one and two fluorophore modifications, respectively. The estimated copy number of DNA loaded per NPs for sequence A, B, and C was ~3.4, ~4.5, ~1.5 times higher than that of sequence D, respectively. Interestingly, Raman intensity of FRNPs derived from sequences B and C exhibited 1.79, 2.30, 4.01 times higher intensity respectively compared to NPs derived from sequence A at the 796 cm^−1^ peak (Fig. 3d). Although FRNPs derived from sequence D had the lowest loading out of the four sequences, it demonstrated the highest SERS intensity. Therefore, we selected sequence D modified with two Dylight^TM^ 780 fluorophores for downstream optimization.

### Plasmonic core selection

**Fig. 4:**
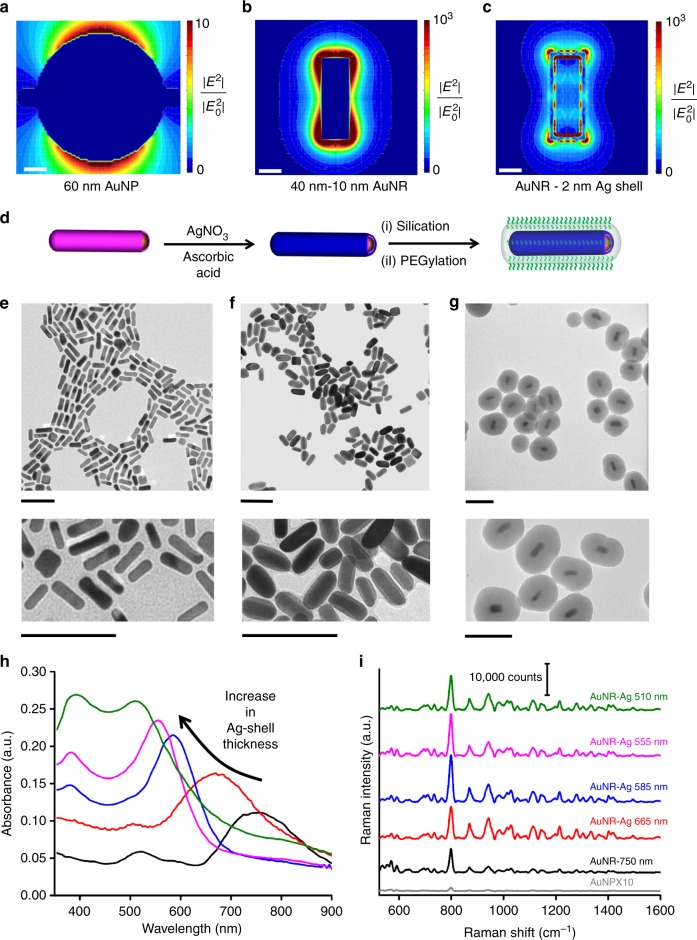
Plasmonic core and shell selection. Electric field enhancement maps of different plasmonic cores: **a** 60-nm AuNP, **b** 40-nm length–10-nm diameter AuNR, and **c** AuNR with 2 nm gap and 2 nm Ag shell. The average electric field enhancement factor is increased by 2 orders of magnitude predicting a higher SERS enhancement with a AuNR core and a Ag shell. Scale bars are 10 nm. **d** Synthesis of the optimized FRNPs. Different thicknesses of Ag shell are formed by reducing different concentrations of AgNO_3_ with ascorbic acid in the presence of DNA cloaked AuNR cores. **e**–**g** TEM images (lower panels are magnified views) of fluorophore cloaked AuNR cores, AuNR core with a 2 nm Ag shell and fully optimized FRNPs with PEG-2000 coated silica shell. All the scale bars are 100 nm. **h** UV–vis spectra of the AuNRs with increasing thickness of silver shell showing a hypsochromic shift of localized surface plasmon resonance (LSPR) peaks. **i** Fluorescence background subtracted Raman spectra of 10 fM of the spherical AuNP-based FRNPs and 1 fM of AuNR-based FRNPs with increasing thickness of Ag. The FRNP with longitudinal LSPR maximum of 585 nm exhibits the maximum Raman enhancement

Theoretically, SERS enhancement is proportional to the local electric field enhancement to the power of 4 (E^4^) at low Stokes shift^39^. Therefore, NPs with local E-field enhancement at the excitation wavelength of 785 nm would produce considerably higher SERS enhancement. To select the plasmonic core for maximum electric field enhancement at 785 nm excitation, we carried out extensive electromagnetic (EM) calculations using discrete dipole approximation (DDA) (see SI for details). The predicted electric field enhancement of an AuNR (10 nm by 40 nm) was ~136-fold higher than that of a 60-nm spherical AuNPs at distances lower than 2 nm (Fig. 4a, b). Inspired by the theoretical predictions, we replaced the 60 nm AuNP cores with AuNRs with an aspect ratio of 4 (*λ*_max_ ≈ 750 nm). We observed ~70 times brighter SERS intensity at the 796 cm^−1^ peak due to the replacement (Fig. 4i).

### Plasmonic shell selection

Previously, it has been reported that the formation of a plasmonic shell onto the DNA coated AuNPs enhances the SERS intensity^46^. Therefore, we extended our EM calculations to predict if the SERS enhancement could be further increased. Encouragingly, theoretical calculations predicted that formation of a 2 nm Ag shell around fluorophore-cloaked AuNRs would increase the local field enhancement and therefore the SERS intensity would increase by the factor of 2.5 (Fig. 4c). Bolstered by the theoretical predictions, we created different thicknesses of Ag shell by adding different amounts of silver precursor and reducing it with ascorbic acid in the presence of the same concentration of fluorophore-cloaked AuNRs (see Methods section for details, Fig. 4d). Upon the formation of different thicknesses of Ag shells, the diameters of the AuNRs were slightly increased as verified by TEM imaging (Fig. 4f and Supplementary Figs. 3, 4). We conducted energy dispersive spectroscopy (EDS) that confirmed the presence of silver after the formation of the silver shell (Supplementary Fig. 5). Increasing thickness of Ag-shell induced a dramatic hypsochromic shift in the longitudinal and transverse LSPR peaks (Fig. 4h). We found that NPs with the longitudinal *λ*_max_ of 585 nm produced ~2-fold and ~134-fold brighter Raman intensity at 796 cm^−1^ compared to the AuNR and AuNP cores, respectively (Fig. 4i).

### Surface passivation and characterization

To ensure in vivo stability, long circulation time, lower macrophage uptake, and increased tumor uptake, we incorporated a surface passivating layer of polyethylene glycol (PEG)^47,48^. First, we considered directly PEGylating the silver shell with thiolated PEGs (MW 2000). However, we observed silver shell decomposition and disappearance of Raman signatures upon serum exposure at 37 °C (Supplementary Fig. 6). Therefore, we created a silica shell and attached PEG-2000 chains via a straightforward NHS ester chemistry (see Methods section for details). The optimized FRNPs (abbreviated as OFRNPs hereafter, major axis 97 ± 5 nm and minor axis 80 ± 7 nm) with AuNR cores are clearly visible in the TEM images (Fig. 4g). The overall hydrodynamic diameter was 138 nm (polydispersity index, *Đ* 0.25) and ζ-potential was −3.25 mV. We further assessed the serum and photo-stability of the surface passivated FRNPs.

### In vitro cell uptake studies

**Fig. 5:**
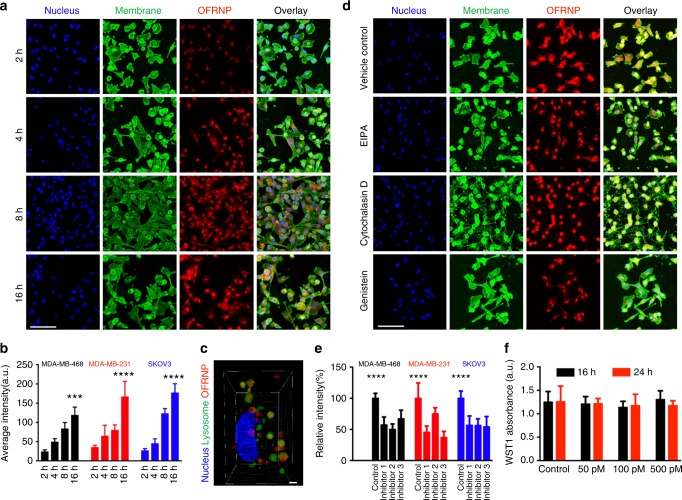
In vitro uptake and toxicity of OFRNPs. **a** Fluorescence microscope images of MDA-MB-231 cells incubated with 100 pM of OFRNPs at different incubation times of 2, 4, 8, and 16 h. Longer incubation time increase uptake of the OFRNPs shown as red. Cell nucleus and membrane were stained with Hoechst® 33342 and wheat germ agglutinin, AF488 conjugate respectively. **b** Quantification of intracellular fluorescence of MDA-MB-468 (black), MDA-MB-231 (red), and SKOV-3 (blue) cells at different durations of incubation with 100 pM of OFRNPs. Scale bars are 100 µm. **c** After 16 h of incubation time, OFRNPs were localized into the cytoplasm in the lysosomal compartments labeled in green. Scale bar is 1 µm. **d** Fluorescence microscope images of MDA-MB-231 cells incubated with 100 pM of OFRNPs in the presence of different endocytosis inhibitors, 75 μM EIPA (inhibitor 1), 10 μg/mL Cytochalasin D (inhibitor 2), 2 μM Genistein (inhibitor 3). **e** Quantification of relative fluorescence intensity of MDA-MB-468 (black), MDA-MB-231 (red), and SKOV-3 (blue) with respect to vehicle control demonstrates diminished endocytosis. **f** WST1 cell viability assay of MDA-MB-231 cells incubated with 500, 100, and 50 pM of OFRNPs exhibits no cell toxicity of the NPs. All data in (**b**, **e**) represent mean ± standard deviation (*n* = 10), ****P < 0.0001, ***P < 0.001 (one-sided Student’s *t* test). All data in (**f**) represent mean ± standard deviation (*n* = 3)

We carried out fluorescence-based in vitro uptake assays of the PEG-2000 coated OFRNPs using the NIR fluorescence. We incubated three different human cancer cell lines MDA-MB-231 (breast), MDA-MB-468 (breast), SKOV-3 (ovarian), and a non-tumorigenic human epithelial cell line MCF10A with 100 pM of OFRNPs at 37 °C. We identified no significant uptake in MCF10A cells compared to MDA-MB-231 cells after 16 h of incubation demonstrating specific uptake by cancer cells (Supplementary Fig. 7). Next, we conducted a time-dependent uptake of the OFRNPs in three different cancer cell lines. We detected a gradual increase in the intracellular fluorescence intensity of the NPs in MDA-MB-231 cells (Fig. 5a) and two other cancer cell lines (Supplementary Figs. 8, 9). We quantified the intracellular fluorescence intensity and found that the intracellular NP concentration increased monotonically with time in all three cell lines (Fig. 5b). We identified that after 16 h of incubation, OFRNPs were localized in lysosomes (as stained with a well-known lysosome marker, LysoTracker® Green DND-26; Fig. 5c). To identify the mechanism of OFRNP uptake, we carried out a fluorescence-based uptake assay in the presence of three well-established endocytosis inhibitors; namely a macropinocytosis inhibitor (5-(N-ethyl-N-isopropyl)amiloride, EIPA), an actin polymerization inhibitor (cytochalasin D), and a caveolae-dependent endocytosis inhibitor (genistein). We found that all the inhibitors were able to significantly decrease intracellular NP concentrations and induce significant morphological changes in MDA-MB-231 cells (Fig. 5d). We observed a similar trend for the other two cancer cell lines (Supplementary Figs. 10, 11). Again, we quantified the intracellular fluorescence and found that OFRNP uptake was decreased by ~50% upon the exposure to the endocytosis inhibitors (Fig. 5e). To test if the cellular uptake of the NPs caused any deleterious effects, we carried out a WST1-based cell viability assay in MDA-MB-231 cells. We did not observe any significant cytotoxicity with three different concentrations of OFRNPs (500, 100, and 50 pM) at 16 and 24 h (Fig. 5f) and the results were cross-verified with Live-Dead assay (Supplementary Fig. 12).

### In vivo dual-mode cancer imaging and photothermal therapy using OFRNPs

Encouraged by the excellent serum and photo-stability and nonexistent cytotoxicity profile in vitro, we explored in vivo imaging capabilities of the OFRNPs. We expected PEG functionalized OFRNPs to accumulate in tumors via passive targeting or the enhanced permeability and retention (EPR) effect^49^. To assess the efficacy of the OFRNPs for cancer imaging, we utilized a subcutaneous ovarian cancer xenograft model and an RCAS/TVA glioblastoma (GBM) mouse model. We chose these cancer models because they present some of the highest mortality rates after diagnosis, and their primary line of treatment is surgical removal of the tumor. Therefore, resection of the exact extent of the tumor is critical for improving the survival rate.

**Fig. 6:**
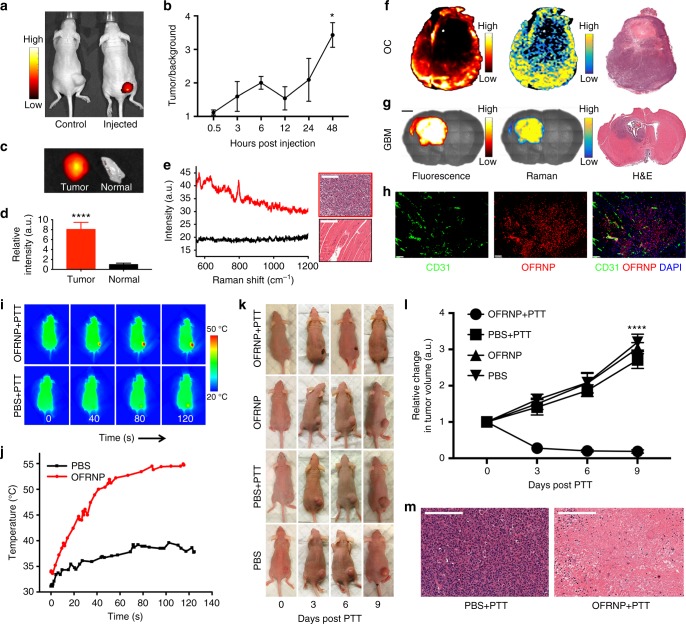
In vivo cancer imaging and photothermal therapy using OFRNPs. **a** NIR fluorescence images of ovarian cancer xenograft mouse after 48 h i.v. administration of 200 µl of 1 µM of Dylight^TM^-780 labeled DNA (left, control), and 200 µl of 10 nM of OFRNPs (right). There is visible contrast in tumor tissue. **b** Tumor to background ratio derived from NIR fluorescence images at different time points. The ratio reaches a maximum of 3 after 48 h post-injection (*n* = 3). **c**, **d** Ex vivo fluorescence images of tumor showing ~8 times higher fluorescence than muscle next to the tumor (*n* = 3). **e** Background subtracted Raman spectra of the tissues indicate the presence of the Raman signature of OFRNPs at 796 cm^−1^ in the tumor tissue but not the normal tissue. Scale bars are 200 μm. **f** Ex vivo fluorescence and Raman images of the excised ovarian cancer tumor with adjacent H&E histology correlation. **g** Ex vivo fluorescence and Raman images of the coronal section of a GBM mouse brain with adjacent H&E histology correlation. Scale bar is 1 mm. **h** High magnification immunofluorescence images of ovarian cancer tumor strained with CD31 antibody (green channel) for the visualization of neovasculature. The OFRNPs (red channel) was clearly localized in and just out of neovasculature. Scale bars are 20 μm. **i** Temperature maps of the mouse during photothermal therapy (PTT) at different laser exposure times. **j** The average temperature of the tumor area during photothermal therapy indicated OFRNP injected mouse tumors underwent an increase in temperature (red) compared to the PBS injected mouse (black). **k** Representative photographic images of a mouse from different treatment groups at different days post photothermal therapy. **l** Relative change in tumor volume of different treatment groups (*n* = 6) plotted against time (in days) after PTT, circle, square, triangle, inverted triangle corresponds to OFRNP injected mice with PTT, PBS injected mice with PTT, OFRNP injected mice without PTT, and PBS injected mice without PTT, respectively. The tumor sizes increase considerably for all the groups, in contrast to size decrease of OFRNP injected mice which received PTT. **m** H&E staining shows extensive necrosis of OFRNP injected tumor compared to PBS injected tumor after the same PTT. Scale bars are 200 μm. All data represent mean ± standard deviation (*n* = 6), ***P < 0.001, *P < 0.05 (one-sided Student’s *t*-test)

We administered 200 µl of 10 nM PEGylated OFRNPs in ovarian cancer xenograft model (*n* = 5) via tail vein injection (see Methods section for details). We carried out fluorescence imaging at different time points post injection. We observed significant fluorescence contrast in tumors compared to normal tissue as early as 3 h post-injection. The presence of OFRNPs could be detected in the blood up to 24 h post-injection (Supplementary Fig. 13). On the contrary, mice injected with 200 µl of 1 µM of fluorophore-labeled DNA did not show any detectable accumulation in the tumor even after 48 h (Fig. 6a, b). The whole tumor can be sequentially and completely resected using fluorescence imaging as visual guidance (Supplementary Fig. 14). We also resected a piece of normal tissue next to the tumor and other vital organs for ex vivo fluorescence and Raman imaging. We found that although the tumor/normal tissue fluorescence intensity ratio was ~8, there was considerable autofluorescence from normal tissue (Fig. 6c, d). Next, we verified that the tumor tissue had the Raman signature of Dylight^TM^ 780 during Raman scanning while the normal tissue exhibited no such signature (Fig. 6e). Quantitative biodistribution experiments (*n* = 3, 24 h post-injection) were carried out by quantifying Au content in the tissue using atomic absorption spectroscopy (AAS). The results revealed that the tumor, liver, spleen, and kidney are the major organs of NP residence 48 h post-injection (Supplementary Fig. 15). To investigate whether OFRNPs could be utilized for mouse models which more closely recapitulate human disease, we carried out imaging in RCAS/TVA glioblastoma (GBM) mouse models. We verified the presence of tumor 6 weeks post-inoculation by MRI (Supplementary Fig. 16a). Tumor-bearing mice were injected with 200 µl of 10 nM of PEGylated OFRNPs and the whole brain was excised 48 h post-injection. Fluorescence imaging of the whole brain revealed a hyperintense area corresponding to the presence of a tumor (Supplementary Fig. 16b).

To correlate the intratumoral distribution of the OFRNPs with the histological status, we carried out scans of excised ovarian tumors with an InVia Raman microscope and processed the data for fluorescence and Raman images as described previously. Ex vivo fluorescence and Raman images showed excellent correlation with the H&E histology images (Fig. 6f). The hypo-intense region in the center of the tumor was corroborated with the necrotic region in the H&E histology images. In the RCAS/TVA GBM mouse models, we carried out scans of a coronal section of the brain. We found that only a part of the brain was fluorescent and Raman positive which correlated very well with the tumors in H&E histology images (Fig. 6g). We also carried out a higher magnification immunofluorescence imaging of the ovarian cancer tumors to corroborate the location of the NPs within the microscopic features. Indeed, we observed the NPs inside and bursting out from the neovasculature labeled with CD31 (Fig. 6h).

Inspired by the excellent in vivo uptake by cancer tissue, we carried out image-guided PTT experiments. We injected 2 groups (*n* = 6) with 200 µl of 40 nM OFRNPs and 2 groups (*n* = 6) with 200 µl of sterile PBS. After 24 h, we irradiated tumors of OFRNP injected, and PBS injected groups with a 660 nm laser (power density of 3 W/cm^2^) for 2 min. We observed a significant increase in temperature in the tumor area of the OFRNP injected mouse exposed to laser irradiation compared to the PBS injected mouse (Fig. 6i, j, Supplementary Figs. 17). We monitored the tumor growth and other vital symptoms for 2 weeks post-injection. We observed that the average tumor sizes of the OFRNP injected group with PTT shrunk by about one-third of the original size. However, the other three groups exhibited tumor growth by a factor of 3 over 10 days (Fig. 6k, l). Histological examination of excised tumors of the four groups revealed that the OFRNP–PTT tumors exhibited extensive necrosis as opposed to the tumors from other treatment groups (Fig. 6m). Also, we observed no change in body weight or abnormal histological changes in major mouse organs in treatment and control groups signifying no major toxicity in mice (Supplementary Figs. 18, 19).

## Discussion

We developed a DNA-based strategy to optimize the functionality of NPs with all-in-one NIR fluorescence and Raman emission for highly efficient cancer optical imaging and PTT applications. We rationally designed the OFRNPs following a step-by-step optimization approach enabled by a fluorophore-modified DNA layer adhered onto the AuNP surface. This approach allowed us to search from an extensive selection of commercially available fluorophores with straightforward attachment chemistry. Theoretically and experimentally surface enhanced resonance Raman scattering (SERRS; excitation laser wavelength matching with the optical absorption of fluorophore) leads to higher enhancement than SERS with non-resonant reporters^50^. We found out that the fluorophore with high absorption cross-section at the excitation wavelength (785 nm), Dylight^TM^ 780 exhibited the maximum Raman intensity compared to other non-resonant fluorophores. Therefore, we selected Dylight^TM^ 780 for the second round of optimization.

The most influential factor in obtaining the maximum SERS enhancement is the surface-fluorophore distance. For spherical NPs, considering only EM enhancement, the SERS enhancement dependence is 1/*r*^12 ^^51^ Therefore it was critical to select a DNA linker which possesses high affinity to gold without distancing the fluorophore from the surface. It has been shown that the relative affinity of nucleobases homo-oligonucleotides to Au surface A>C≥G>T^42,52^. Therefore, we selected a 6-nucleotide long oligo-A sequence with regular phosphate backbone ((PO)A6) and one with modified phosphorothioate ((PS)A6) backbone. We also selected a non-adenine sequence ((PO)TCGCGC) as a negative control. To verify the effect of the additional sulfur moieties on the overall orientation of DNA molecules on AuNP surface, we carried out extensive MD simulations. We found that indeed the fluorophore-modified (PS)A6 sequence demonstrated lying down orientation on the AuNP surface and, therefore the fluorophore-surface distance was slightly lower than that of (PO)A6 DNA and the control sequence. The insightful information from the MD simulations led us to select four different DNA sequences (A–D, Fig. 3c) to test our hypothesis. Interestingly, we found the sequence D, (PS)A6 with two Dylight^TM^ 780 modifications demonstrated the highest Raman intensity at 796 cm^−1^ despite being loaded at 2–3 times lower density onto the AuNP surface. MD simulations predicted that (PS)A6 would create lower fluorophore-AuNP surface distances compared to the (PO)A6 DNA. Therefore, sequences C and D possessed higher Raman signal despite the lower loading density. However, sequence D was labeled by two fluorophores/DNA compared to sequence C (one fluorophore/DNA). Therefore, sequence D demonstrated the highest Raman intensity amongst all the sequences. This result suggested the minimization surface-fluorophore distance has a higher impact on SERS enhancement compared to the surface density.

In the next level of optimization, we selected the plasmonic NP core that further enhances the SERS intensity. DNA-based design of plasmonic core allowed us to select any of the shapes and sizes from a huge selection of plasmonic NPs that can be synthesized by solution-based chemistry. From previous theoretical and experimental calculations, it has been demonstrated that NPs having absorption maxima (plasmonic peak) coinciding with incident light provided higher SERS enhancement. Therefore, AuNRs which have tunable LSPR absorbance maxima spanning 650–900 nm appeared to be an obvious option. We carried out a calculation of electric field enhancement for 60 nm AuNP and 40 nm by 10 nm AuNR using a DDA model. The predicted average electric field enhancement of AuNRs is ~136 times higher than that of AuNPs. Further, the electric field can be enhanced by 1.5 times just by growing a thin silver shell on top of the DNA layer. The theoretical inference led us to replace AuNPs with AuNRs as the plasmonic core. Experimentally we observed 70-fold brighter SERS intensity for AuNR based core compared to the AuNP based core. Next, we formed a thin silver shell on the DNA shell by reducing silver precursors with a reducing agent. The formation of a silver shell was verified by UV–vis spectroscopy, TEM, and EDS elemental analysis. Excitingly, we found out that the SERS signal could be further enhanced by 2 times upon the silver shell formation. We passivated the NP with a silica shell and PEG-2000 layer for in vivo applications. We observed 10–20% drop in SERS intensity, potentially stemming from slight alteration of the fluorophore-surface distances during the functionalization process. The limit of detection of the PEGylated OFRNPs was found to be 5–10 fM which is a million-fold brighter than that of the initial design consideration and is comparable to the limit of detection of the ultra-bright SERS NPs reported from our group^14^. The OFRNPs demonstrated excellent photo- and serum stability in biological conditions.

Because the OFRNP design does not include any specific targeting agent, we could use these OFRNPs for imaging cancers via the EPR mediated uptake mechanism. Although EPR-based uptake was shown to be heterogeneous across different tumors, the efficacy can be easily predicted or enhanced using clinically approved nanodrugs^53,54^. To demonstrate the versatility of the OFRNPs as an imaging agent, we successfully carried out dual mode imaging of two different cancer models known for high EPR effect. In SKOV-3 xenograft cancer models, the OFRNPs started to generate contrast only 3 h post-injection and was stable for several days, highlighting the robustness of the OFRNPs. Both ex vivo fluorescence and Raman imaging demonstrated preferential accumulation of the OFRNPs in cancerous tissue. We further carried out high-resolution dual mode fluorescence-Raman mapping of excised tumor tissues to assess the efficacy to delineate tumors. The Raman and fluorescence maps were in excellent agreement with the H&E histology for both SKOV-3 xenograft and RCAS/TVA mouse models.

Finally, we demonstrated the efficacy of the OFRNPs for image-guided photothermal ablation of tumors. The OFRNPs were found to be highly efficient in ablating the tumor in the presence of NIR laser irradiation of power density 3 mW/cm^2^. However, OFRNPs without any laser irradiation did not influence the tumor growth or pose any toxic side effects in major organs. The major novelty of this work lies in the DNA-based rational design of the OFRNPs which offers effective fluorescence and Raman modalities and could be detected up to the low femtomolar regime. The low limit of detection enabled efficient and high-resolution bimodal imaging of cancer in vivo and ex vivo.

In conclusion, we have carried out a rational design optimization enabled by DNA to work out the optimum design construct for fluorescence and Raman imaging. OFRNPs have shown excellent localization in cancer tissues in two different cancer models. We also demonstrated excellent PTT via ablation of tumors in ovarian cancer xenograft mouse models. We foresee that the current OFRNPs design would open up the possibility of NIR fluorescence and Raman-based cancer imaging and surgery in preclinical settings given that Raman and fluorescence endoscopes are already in clinical trials or clinically approved, respectively, and could potentially be integrated^55^. Also, the PTT therapy can be conducted by the same imaging laser wavelength in the tumor margins to ablate any residual tumor tissue via minimally invasive or endoscopic approaches. This rational design optimization can be further extrapolated and opens up the possibility of forming degradable ultra-small AuNP clusters, aiming at renal clearable OFRNPs which might have higher chances of clinical translation.

## Methods

### Materials

All chemicals of the highest purity were purchased from Sigma-Aldrich (St. Louis, MO) and used as received. All DNA sequences were purchased from IDT DNA Inc. as HPLC purified grade and used without further purifications. NHS ester modified fluorophores, Zeba desalting columns (7k MWCO) were purchased from Thermo Fisher Scientific. G-25 size-exclusion columns were purchased from GE Healthcare.

### Fluorophore labeling of bifunctional DNA

Fluorophore labeling of DNA was carried out by reacting NHS labeled fluorophore with amine-modified DNA molecules (sequences A–D). In a typical reaction, 100 μL of 10 mM NHS modified fluorophore solution in anhydrous N,N-dimethylformamide (DMF) was mixed with 100 μL of 1 mM amine modified DNA in DI water. To the reaction mixture, 20 μL of trimethylamine was added. After 12 h of reaction at room temperature, the reaction mixture was lyophilized and the excess fluorophore molecules were removed using a Zeba Spin desalting column.

### AuNP functionalization with fluorophore-labeled thiolated DNA

The disulfide bonds in the fluorophore-labeled thiol-modified oligonucleotides (1 mM) were reduced to monothiol using 50 mM Tris(2-carboxyethyl)phosphine hydrochloride (TCEP) in water. The reduced oligonucleotide was purified using a G-25 size exclusion column to remove the excess small molecules. The purified monothiol-modified oligonucleotides were incubated with 60 nm AuNPs in a 5000:1 ratio in 0.5× TBE buffer (44 mM Tris, 44 mM boric acid, 1 mM EDTA, pH 8.0) and 0.5% sodium dodecyl sulfate (SDS). The Na^+^ concentration was gradually increased to 350 mM over 24 h by adding 5 aliquots of 5 M NaCl at room temperature under gentle shaking to maximize the surface coverage of the AuNPs by the thiolated DNA. The functionalized DNA was centrifuged (11,000 × *g* for 5 min) and redispersed in 1× TBE buffer with 0.5% SDS, to remove excess oligonucleotides. The concentration of AuNPs was measured using UV–vis spectroscopy.

### AuNR functionalization with fluorophore-labeled thiolated DNA

As synthesized AuNRs were centrifuged and washed with DI water 2 times to remove excess CTAB at 13,000 rpm for 15 min. The AuNRs were dispersed in 0.5× TBE buffer with 0.5% SDS and the purified fluorophore modified DNAs were added in 1:1000 ratio. After 6 h of incubation, a 5 M NaCl solution was slowly added in seven aliquots to bring the final Na^+^ concentration to 350 mM over 24 h. Then the solution was allowed to sit at room temperature overnight. The excess DNA was removed by 2 times centrifugation (11,000 × *g*, 15 min) and re-suspension in 1× TBE buffer with 0.5% SDS. The concentration of AuNRs was measured using UV–vis spectroscopy.

### Silver-shell formation of fluorophore grafted AuNRs

To a 100 μL solution of 6 nM DNA modified NRs in a 1.5 mL Eppendorf tube, 0.5–5 μL of 100 mM AgNO_3_ was added and mixed immediately using vortex. In another Eppendorf tube, 5 μL of 500 mM ascorbic acid was added. Thereafter, the contents of two tubes were mixed rapidly using a pipette. The color of the solution was changed from brown to bluish green color within few seconds of mixing confirming the silver shell formation. The NPs were then characterized using TEM, DLS, and used for further surface modification.

### Surface passivation of FRNPs

To 1.3 mL of 3 nM NP (AuNP or AuNR) solution, 13 mL isopropanol, 500 μL tetraethylorthosilicate (TEOS), and 200 μL of ammonium hydroxide (NH_4_OH) were added in a 50 mL Falcon tube. The mixture was gently mixed in a shaker for 12 min to form a uniform silica shell around the NPs. The mixture was centrifuged and washed with ethanol for 3 times. The silicated FRNPs were functionalized with 2000 Dalton PEG in a series of steps. In the first step, the silica surface was functionalized with amine groups. To a 1 mL, 10 nM silicated FRNPs in ethanol, 100 μL of 3-aminopropyltrimethoxysilane (APTMS) was mixed at ambient temperature. After 1 h of reaction, the particles were washed 2 times with ethanol and 1 time with DI water. In the next step, the aminated NPs were PEGylated using a PEG2000-NHS ester. To a 1000 μL, 10 nM NP solution in 10 mM sodium bicarbonate buffer (pH 8.6), a 100 μL of 1 mM PEG2000-NHS solution was added and kept for gentle shaking for 2 h in the dark at room temperature. Next, the particles were washed with water 3 times and redispersed in 10 mM MES buffer (pH 7.4) for characterization and in vitro and in vivo studies.

### Characterization of FRNPs

The FRNPs were characterized using a transmission electron microscopy (JEOL 1200, 120 keV, ×80,000–150,000 magnification). The size and concentration of the SERS NPs were measured on a Nanoparticle Tracking Analyzer (Malvern Instruments, Malvern, UK). DLS data was measured using Zetasizer Nano ZS (Malvern Instruments). Raman spectra were obtained in an InVia system equipped with a 785-nm laser (Renishaw Inc., Hoffman Estates, IL).

### Estimation of the grafting density of DNA

The number of DNA molecules attached to the NP was measured using a fluorescence-based assay. The fluorescence intensity at 780 nm of Dylight^TM^ 780 modified DNA was measured before and after the incubation with NPs. The difference between the fluorescence intensity is proportional to the number of the DNA attached to the particle. We determined the numbers of DNA by comparing the difference with the fluorescence intensity of known concentration of the same DNA.

### Serum and photo-stability

Extensive in vitro serum and photo-stability studies of the OFRNPs were carried out. OFRNP was incubated with 50% mouse serum at 37 °C over 16 and 24 h and the fluorescence and Raman intensity was measured over time. A decrease of ~26% in fluorescence intensity at 815 nm and ~27% decrease in Raman peak intensity at 796 cm^−1^ was observed (Supplementary Fig. 20). A 10 pM solution of FRNPs was exposed with the 785-nm laser at three different powers (1.6, 8.2, and 15.8 mW) for 10 min. A ~30% decrease in the fluorescence intensity at the highest laser power was observed. Raman peak intensity at 796 cm^−1^ did not change over time demonstrating robust photostability of the Raman modality (Supplementary Fig. 21). The hydrodynamic diameter and zeta potential were changed by 12.8 nm and 13 mV, respectively upon serum exposure due to adherence of serum proteins to FRNP surface (Supplementary Fig. 22). Therefore, OFRNPs demonstrated excellent serum and photo-stability, required criteria for bioimaging applications.

### Bimodal imaging of tissue-phantom

To demonstrate the dual-mode imaging capability, a tissue phantom with a 100 fM of OFRNPs was created. The phantom was imaged using the same setup for the ex vivo imaging with an InVia Raman microscope (Renishaw, 161 mW laser power, with 1.5 s acquisition time, using the StreamLine high-speed acquisition mode). After the point-by-point data acquisition, the data was processed using a graphical user interface developed in-house to plot the total intensity at Raman shift 600 cm^−1^ (815 nm) to obtain an image corresponding to the fluorescence signal (Supplementary Fig. 23a). Further, the point-by-point spectra were background subtracted to remove the broad fluorescence background signal and give rise to purely Raman spectra. The intensity at Raman shift 796 cm^−1^ was plotted to create the Raman image at that particular wavenumber (Supplementary Fig. 23b). To fully exploit the specificity of the Raman fingerprint, the same data was converted to a map using a direct classical least-squares (DCLS) model which identified the presence of a particular spectral signature (Supplementary Fig. 24a). The limit of detection of these NPs was found out to be ~5 fM, using the same imaging parameters used for in vivo imaging (Supplementary Fig. 24b).

### Estimation of OFRNP limit of detection

In order to determine the limit of detection of the OFRNPs, desired concentrations with serial dilutions were mixed in 1% agarose and cast in a 1536 well plate. For the limit of detection in blood experiments, mouse blood was collected using a syringe from the tail artery of a healthy mouse. As an anticoagulant, 10 µL of 500 mM EDTA solution was added to 1 mL of blood (final concentration of ~5 mM). A serial dilution of the OFRNPs was carried out using blood and pipetted the NP of different concentrations onto a 1536 well plate. The plates were scanned using the exact setup used for the actual tumor imaging (100% laser power, 1.5 s integration time, ×5 objective). For the analysis of the acquired images, a graphical user interface using MATLAB (R2014b) and PLS Toolbox v.8.0 (Eigenvector Research, Inc., Wenatchee, WA, USA) developed in-house was used.

### Cells and cell culture

All cell lines were purchased from ATCC. SKOV-3 (ATCC^®^ HTB-77^TM^) and MDA-MB-231 (ATCC^®^ HTB-26^TM^) cell lines were maintained in RPMI-1640 media and MDA-MB-468 (ATCC^®^ HTB-77^TM^) cell line was maintained in Dulbecco’s modified Eagle’s medium/Ham’s F-12 in humidified incubators at 37 °C. The DF-1 cell line was maintained in DMEM medium. All cell culture media was supplemented with 10% heat-inactivated fetal calf serum, 2 mM L-glutamine, penicillin (20 U mL^−1^), and streptomycin (20 μg mL^−1^).

### Cell viability studies

*WST1 assay*: WST-1 assay was carried out using Cell Proliferation Reagent WST1 (Roche). In brief, ~10,000 cells were seeded in a tissue culture treated 96-well microplate. The cells were incubated with different concentration of OFRNPs for 16 h. For positive control, cells were treated with 70% Methanol for 2 h. After the incubation, 10 μL of WST1 reagent was added. After 2 h of incubation at 37 °C, the UV–vis absorbance of the well was measured at 440 nm. The intensity at 440 nm was compared with the untreated control to determine the cell viability.

*Live dead assay*: Live-dead assay was carried out using a commercially available kit (LIVE/DEAD® viability/cytotoxicity kit, Invitrogen). In brief, cells were seeded in 4-well microscopy slides in 20,000 cells/well concentration. Then, cells were incubated with 1 pM OFRNPs for 16 h before the live-dead staining. The working concentration of live stain Calcein AM was 2 μM and dead stain EthD-1 was 4 μM. We used 70% methanol for 30 min for a positive control. After 30 min incubation at room temperature, the viability was verified using a fluorescence microscope using 495 nm excitation and 515 nm emission for Calcein AM and 635 nm emission for EthD-1.

*Confocal microscopy*: Confocal microscopy of the fixed cells was done in Leica-SP8 microscope. Cells were fixed in 4% paraformaldehyde and stained with Hoechst® 33342 (1 μg/mL) and wheat germ agglutinin AF488 (5 μg/mL) for nucleus and cell membrane respectively prior to imaging. Images were processed and quantified using Imaris 9.1.2 (Bitplane) software.

### Animal studies

All animal experiments were approved by the Institutional Animal Care and Use Committees of Memorial Sloan Kettering Cancer Center. We have complied with all relevant ethical regulations.

### Tumor models

For subcutaneous ovarian cancer tumors, 8-week-old female outbred homozygous nude mice (Foxn1^nu^, Jackson Laboratory) were subcutaneously injected with 2 × 10^6^ SKOV-3 (HTB-77, ATCC) suspended in 0.2 mL culture media mixed with 0.2 mL of Matrigel (Corning) into the lower back side. We waited for 2 weeks post inoculation or until tumor sizes reached 0.5 × 0.5 cm.

The well-established somatic gene transfer system RCAS/tv-a was utilized to generate the GBM-bearing mice which closely recapitulates human GBMs^19,56^. 4–8 weeks old *Nestin*-tv-a/*Ink4a-arf*^*−/−*^/*Pten*^*fl/fl*^ mice were used. Mice were anesthetized with intraperitoneal injections of ketamine (0.1 mg/g) and xylazine (0.02 mg/g) and injected with 4 × 10^4^ DF-1 cells (CRL-12203, ATCC) transfected with RCAS-*Pdgfb* and RCAS-Cre (1:1 mixture, 1 µL) into the brain using 30-gauge needle attached to a Hamilton syringe. Injections were targeted to the subventricular zone, coordinates bregma 1.7 mm (anterior), Lat 0.5 mm (right), and depth 2.5 mm from the dural surface. The genetic aberrations such as overexpression of oncogene *Pdgfb* and loss of tumor suppressor genes (*Ink4a-arf* and *Pten*) led to the formation of glioblastoma within 2 months. Tumor incidence and size were determined by weekly MRI scans (Bruker Biospin Corp., Billerica, MA) starting 4 weeks after injection.

### Fluorescence and Raman imaging

Mice were administered 200 μL of 10 nM OFRNPs in 10 mM MES buffer (pH 7.1–7.3) via tail vein injection. Mice were anesthetized with isoflurane for in vivo imaging or euthanized with CO_2_ and then dissected for ex vivo imaging. Fluorescence images were acquired with an IVIS-200 imaging system (Xenogen Corp., Hopkinton, MA). Radiance (photons/s^−1^ cm^−2^) was calculated for the region of interest using Living Image V4.2 software.

### Ex vivo Raman imaging

All Raman scans were performed on an InVia Raman microscope (Renishaw) equipped with a piezo-controlled stage for spatial mapping, a 300-mW 785-nm diode laser and a 1-in. CCD detector with a spectral resolution of 1.07 cm^−1^. The SERS spectra were acquired through a ×5  objective lens (Leica). Typically, Raman scans were carried out at 100 mW laser power, with 1.5 s acquisition time, using the StreamLine high-speed acquisition mode. All Raman images were acquired and analyzed under the same conditions, including the same laser power, Raman integration times, and focal volume (same objective lens).

### Biodistribution of OFRNPs

Homogenized tissue specimens (*n* = 3) of known mass were put in Savillex PFA vials with 1 mL of freshly prepared aqua regia (1:3 conc. nitric acid and hydrochloric acid) and heated for 12 h at 60 °C for complete digestion. When the samples fully dissolved in the acidic solution, 100 μL of the digest were taken up in 15 mL Falcon tubes and the volume was made up to 5 mL using deionized water and analyzed with an atomic absorption spectroscope and compared with HAuCl_4_ calibration solutions of known concentration.

### Histology

After imaging, multiorgan specimens were fixed in 4% paraformaldehyde (MP Chemicals, Solon, OH, USA) overnight at 4 °C, followed by a rinse with PBS for 15 min, and then kept in 70% ethanol until embedding in paraffin. Five-micrometer-thick sections were cut from the paraffin block and stained with H&E and scanned with a Mirax digital slide scanner (Zeiss, Jena, Germany) for histopathological analysis. The slides were analyzed with Pannoramic Viewer software (3DHistech, Hungary).

### Reporting summary

Further information on experimental design is available in the Nature Research Reporting Summary linked to this article.

## Supplementary information

Supplementary Information

Description of Additional Supplementary Files

Supplementary Movie 1

Supplementary Movie 2

Supplementary Movie 3

Reporting Summary

## Data Availability

Custom codes related to the graphical user interface using MATLAB (R2014b) and PLS Toolbox v.8.0 (Eigenvector Research, Inc., Wenatchee, WA, USA) developed in-house are available upon reasonable request.

## Data availability

The data that support the findings of this study are available from the authors upon reasonable request, see author contributions for specific data sets.
