# Supplementary material for: DNA-enabled rational design of fluorescence-Raman bimodal nanoprobes for cancer imaging and therapy
Source: Nat Commun. 2019 Apr 26;10:1926. doi: 10.1038/s41467-019-09173-2 (PMC6486596; doi:10.1038/s41467-019-09173-2)
Supplement: Supplementary file 1 — Supplementary Information [file 41467_2019_9173_MOESM1_ESM.pdf]

Supplementary Information

DNA-enabled Rational Design of Fluorescence-Raman Bimodal Nanoprobes for Cancer Imaging and Therapy

*Suchetan Pal[1, 2], Angana Ray[3], Chrysafis Andreou[1, 2], Yadong Zhou[4], Tatini Rakshit[5], Marek Wlodarczyk[6], Masatomo Maeda[2], Ricardo Toledo-Crow[7], Naxhije Berisha[1, 2, 6], Jiang Yang[1, 2], Hsiao-Ting Hsu[1, 2], Anton Oseledchyk[1, 2], Jagannath Mondal[3], Shengli Zou[4], Moritz F. Kircher[1, 2, 8 ,9,10 *]*

[1] Center for Molecular Imaging and Nanotechnology (CMINT), Memorial Sloan Kettering Cancer Center, New York, NY 10065, USA

[2] Department of Radiology, Memorial Sloan Kettering Cancer Center, New York, NY 10065, USA

[3] Tata Institute of Fundamental Research, Hyderabad, Telangana, 500107, India

[4] Department of Chemistry, University of Central Florida, Orlando, FL 32816, USA

[5] Department of Bioengineering, New York University, New York, NY 10010, USA

[6] Ph.D. Program in Chemistry, The Graduate Center of the City University of New York, New York, NY 10016, USA

[7] Advanced Science Research Center, City University of New York, New York, NY 10031, USA

[8] Molecular Pharmacology Program, Sloan Kettering Institute, NY 10065, USA

[9] Department of Radiology, Weill Cornell Medical College, New York, NY 10021, USA

[10] Department of Imaging, Dana Farber Cancer Institute, Boston, MA 02215, USA

[*]Corresponding author. Email: moritz_kircher@dfci.harvard.edu

Supplementary Methods

1. Synthesis of 60 nm AuNP core

The AuNP core was synthesized using a modified protocol using a seed mediated growth method. In a typical synthesis procedure, we first synthesized ~ 15 nm AuNP cores. To 99 ml deionized water, 1 ml 25 mM $HAuCl_4$ was added and the solution was heated on a heating plate to boil. To the boiling solution 1 ml of 3.3% sodium citrate solution was added. After 15 minutes the color of the solution had changed to red confirming the formation of 15 nm AuNP cores. In the next step, to 100 ml deionized water 125 µL of 200 mM $HAuCl_4$, 30 µL 500 mM trisodium citrate and 700 µL 15 nm AuNP cores were added under stirring. Further, 250 µL of 1M hydroxylamine hydrochloride solution was mixed. Within few seconds the color of the solution turned deep red confirming the synthesis of 60 nm AuNP.

2. Synthesis of 40 nm-10 nm AuNR core

The synthesis of AuNRs was carried out using the silver-assisted growth procedure from previous report.[1]

a. AuNP Seed synthesis: 60 µL of 10 mM ice cold sodium borohydride solution was mixed to 1 mL of 2.5 mM Hydrogen tetrachloroaurate(III) hydrate solution in 100 mM Cetrimonium bromide (CTAB) and under vigorous mixing. The solution color immediately changed to yellowish brown confirming the formation of small sized seeds that act as nucleation seed for AuNR growth.

b. AuNR synthesis: To 100 mL of 100 mM CTAB solution, 80 µL of 100 mM silver nitrate and 500 µL of 100 mM Hydrogen tetrachloroaurate(III) hydrate was mixed. After gentle mixing, 1200 µL of 100 mM ascorbic acid solution was added and mixed thoroughly. To this mixture, 600 µL of the previously prepared AuNP seed solution was added. The mixture was kept undisturbed at least one hour. The solution turned purple confirming formation of AuNP and verified using UV-Vis spectroscopy and TEM imaging.

3. Molecular dynamic simulation methodology

Gold nanoparticles are known to form face-centered cubic (fcc) lattice with a truncated octahedral motif.[2,3] We have also chosen a truncated octahedral gold nanoparticle composed of 249 gold atoms for this study. The starting structure for gold nanoparticle, Au-55 core (1.2nm), was taken directly from Online JSmol Resources (https://chemistry.beloit.edu/edetc/pmks/pages/gold.html). Further, we developed two single stranded DNAs: (i) ss-DNA (PODNA) and (ii) ss-Phosphorothioate DNA (PSDNA). Both of the single stranded oligonucleotides are composed of six adenine nucleotides, linked to the surface of

the gold nanoparticle by a six-carbon alkyl thiolate linker and tagged with the Lumiprope Cy family dye through linker Lysine.

The initial helical parameters of the PODNA were adapted from the structure of an oligo(dA).oligo(dT) tract.[4] The force field parameters for PODNA are taken from AMBER-ff99bsc0, while the parameters for the alkyl thiol are taken from the work of Hautman and Klein.[5,6,7] The force field parameters for the dye and associated linker (Cy7+Lys) were taken from Graen et. al., and are compatible with AMBER force fields.[8] The interactions between the gold atoms are described by Lennard-Jones potentials where the parameters σ = 2.569 Å and ε = 0.458 eV are taken from the literature.[9] The starting structure of ss-DNA connected with alkyl thiolate linker was developed using MOLDEN software and the PSDNA structure was obtained by replacing O1P-atom (i.e., one of the O-atoms of backbone phosphate group) of PODNA with a S-atom.[10] The initial structure of the Cy7+Lys (dye) system was again taken from Graen et. al.

In each of the simulations, the gold nanoparticle linked and dye-tagged PODNA/PSDNA was explicitly solvated with TIP3P water molecules in a rectangular periodic box whose dimensions were at least 1.5 nm larger than the size of the corresponding solute molecules; and subsequently charge neutralized by adding 4 Na+ ions. The entire set-up was generated using GROMACS 5.0.7.[11] Each system with a cubic box size of 9.084 nm, consisted of ~24363 water molecules and a total of ~73643 particles. The initial round of simulation with explicit solvent and ions involved 50000 steps of steepest descent energy minimization to remove the high-energy contacts. The position of the gold atoms was fixed with harmonic constraints. Thereafter, a 100 ns molecular dynamics simulation at 300 K with a NVT ensemble was performed while keeping the coordinates of gold atoms frozen using 0.001 ps as time step. The particle mesh Ewald (PME) summation method was used to treat long-range electrostatic interactions (with fourier spacing of 0.12 nm and interpolation order 4) and force-switch method was applied for non-bonded interactions (van der Waals) with a cutoff of 1.0 nm.[12] The real-space cut-off was set to 1.0 nm. The verlet cut-off scheme was implemented. We used V-rescale for maintaining the average temperature of 300 K.[13] All the hydrogen atoms bonded to the heavy atoms are constrained using LINCS algorithm to their respective equilibrium bond-length.[14] All the water molecules were simulated as rigid molecule using SETTLE.[15] Both the systems (PODNA and PSDNA) were individually simulated multiple times by varying initial velocity distributions of the systems. Atomic coordinates were saved every 0.5 ps for the trajectory analysis.

We have also performed control simulations with the sequence 5'-TCGCGC-3'. The initial helical parameters of this PODNA were adapted from the structure of Dickerson Drew Dodecamer and the corresponding PSDNA structure was obtained by replacing O1P-atom of PODNA with a S-atom.[16] The simulation protocol was exactly the same as described here in the method section for the A6-sequence. The PSDNA and PODNA systems were each simulated multiple times independently with different initial seeds, reaching a cumulative of 1 μs long MD-run.

All the trajectories were analyzed with GROMACS 5.0.7 and the movies are prepared with VMD.[17]

Electromagnetic simulation methodology

We calculated the electric field near metal nanoparticles using the discrete dipole approximation method. [(45) Draine, B. T. The Discrete-Dipole Approximation and Its Application to Interstellar Graphite Grains. The Astrophysical Journal 1988, 333, 848-872]. The dielectric constants of Ag and Au were both obtained from the handbook of Palik. [Palik, E. D., Handbook of Optical Constants of Solids; Academic press, 1998; Vol. 3]. In the figures, the values of $|E|^2$ are used. The Raman enhancement factor not only depends on shape, also depends on resonance wavelength and Raman excitation wavelength. For the Raman enhancement calcualtions, we used the equation $|E|^4$.

Supplementary Figures

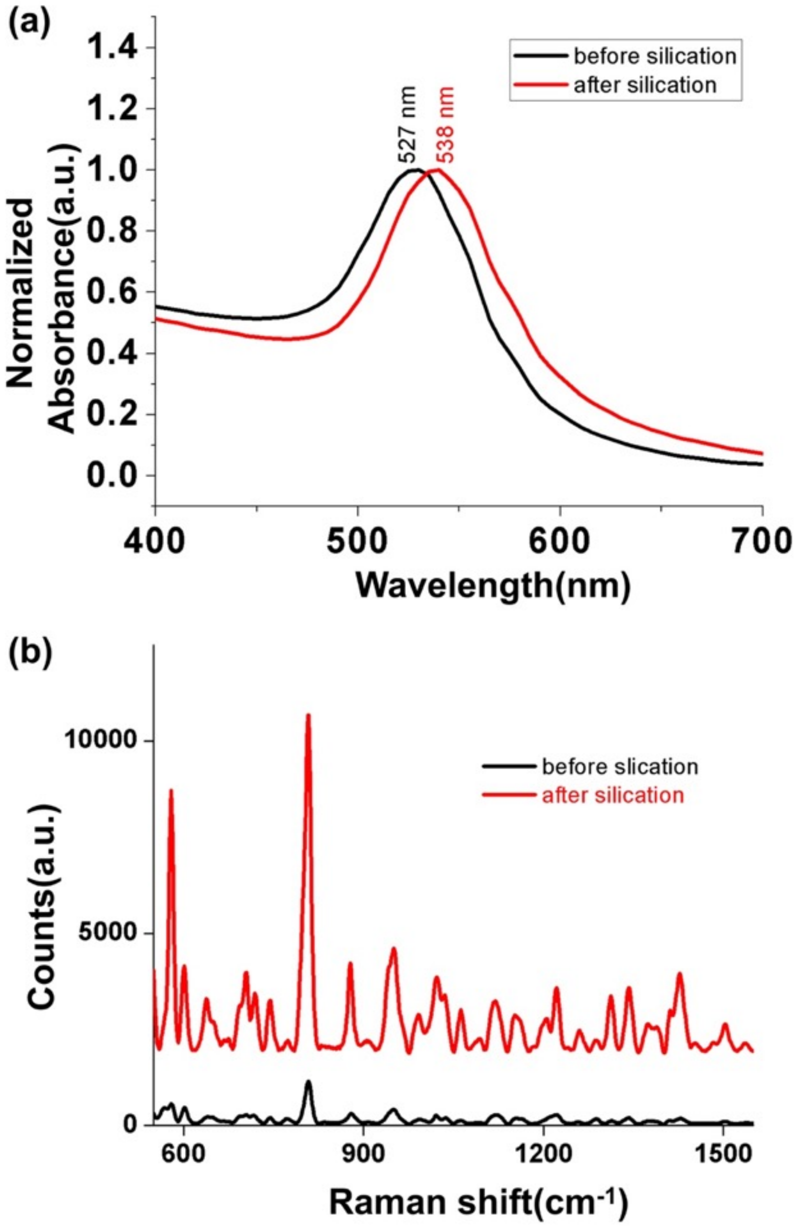

Supplementary Figure 1. (a) UV-Vis spectra of 60 nm AuNP-core based FRNPs before (black) and after (red) silication. We observed ~9 nm bathochromic shift of the absorption maximum. (b) Fluorescence background subtracted Raman spectra of same concentration of FRNPs before (black) and after (red) silication. After silication the Raman peak at 796 cm^{-1} 10 times higher due to decreased surface-fluorophore distance and change in dielectric environment.

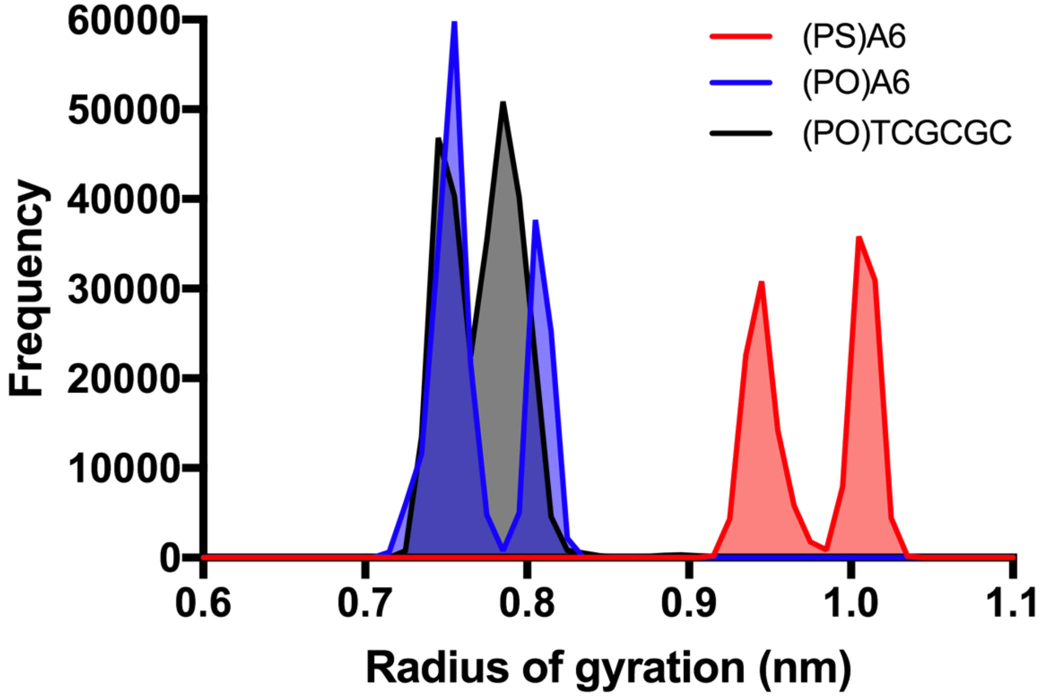

Supplementary Figure 2. The frequency distribution of radius of gyration of HS-(PO)A6-Cy7 (blue), HS-(PS)A6-Cy7(red) and HS-(PO)TCGCGC-Cy7 (black) attached to a 1.2 nm AuNP in the lowest energy conformation in MD simulations. The average radii of gyration of HS-(PO)A6-Cy7 (0.77±0.02 nm) and HS-(PO)TCGCGC-Cy7 (0.77±0.02) are significantly lower than that of HS-(PS)A6-Cy7 (0.97±0.03 nm) and indicating PSA6-Cy7 DNA brings the fluorophore closer to the NP surface.

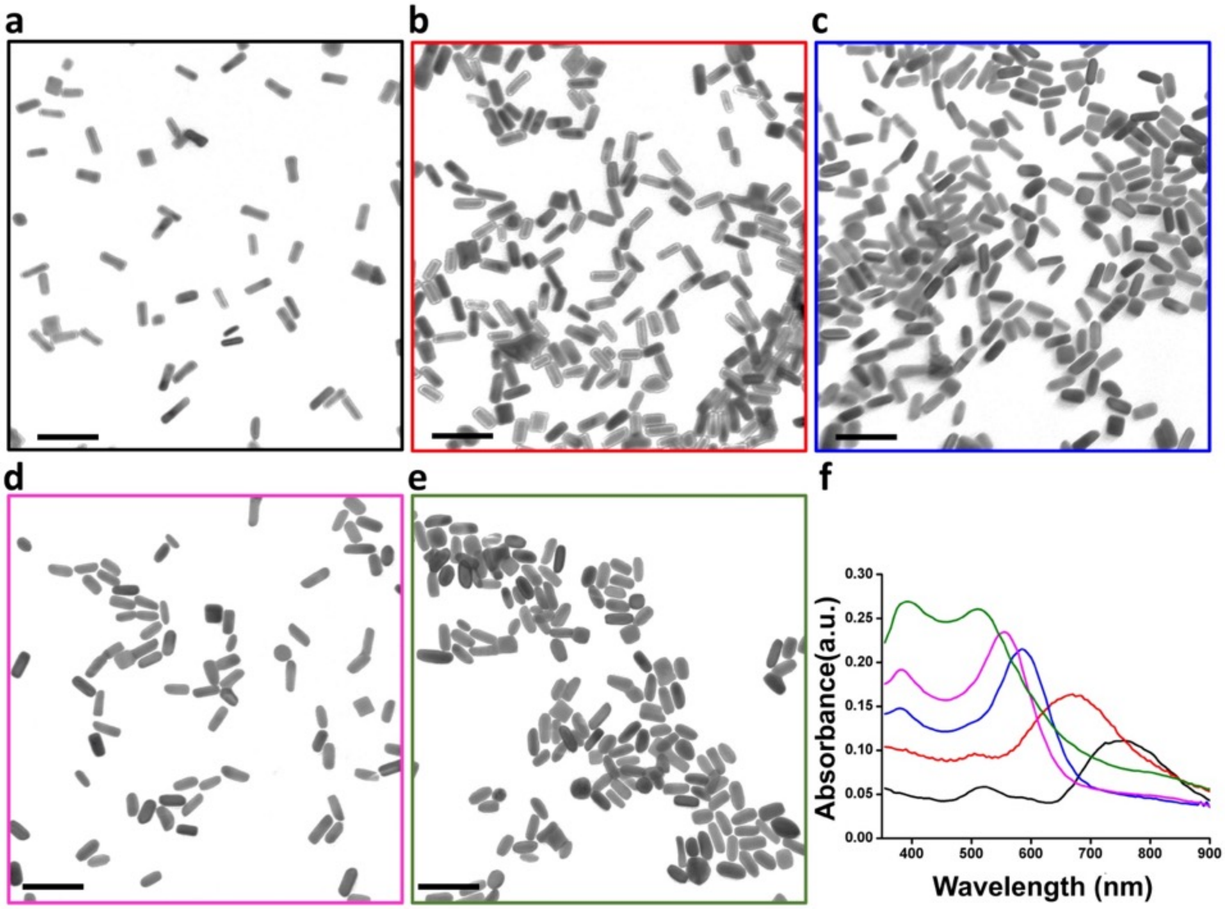

Supplementary Figure 3. Representative TEM images of (a) AuNR and (b-e) Ag coated AuNRs with λ_{max} of the longitudinal SPR of 665 (red), 585 (blue), 555 (pink) and 510 (green) nm respectively.

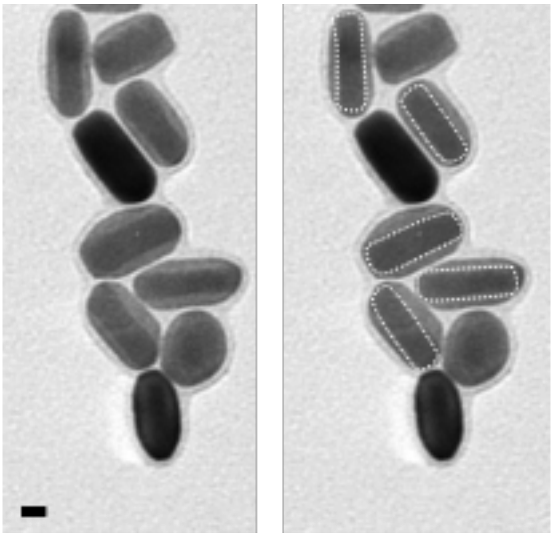

Supplementary Figure 4. A high magnification TEM image of AuNR-Ag 585 showing about 2-5 nm thick Ag shell is formed around a 10nm-40 nm AuNR. Scale bar is 10 nm.

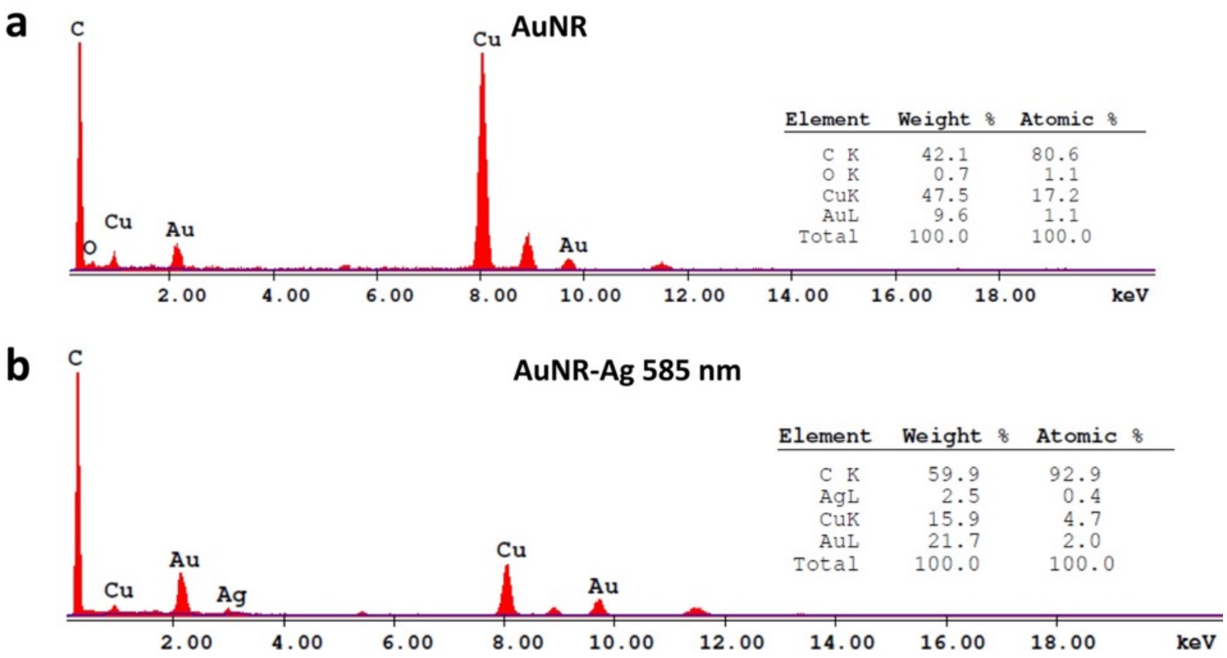

Supplementary Figure 5. Energy dispersive X-ray spectroscopy (EDX) of (a) AuNR and (b) AuNR-Ag 585 nm showing the presence of Ag. C and Cu signal was observed due to the TEM grid.

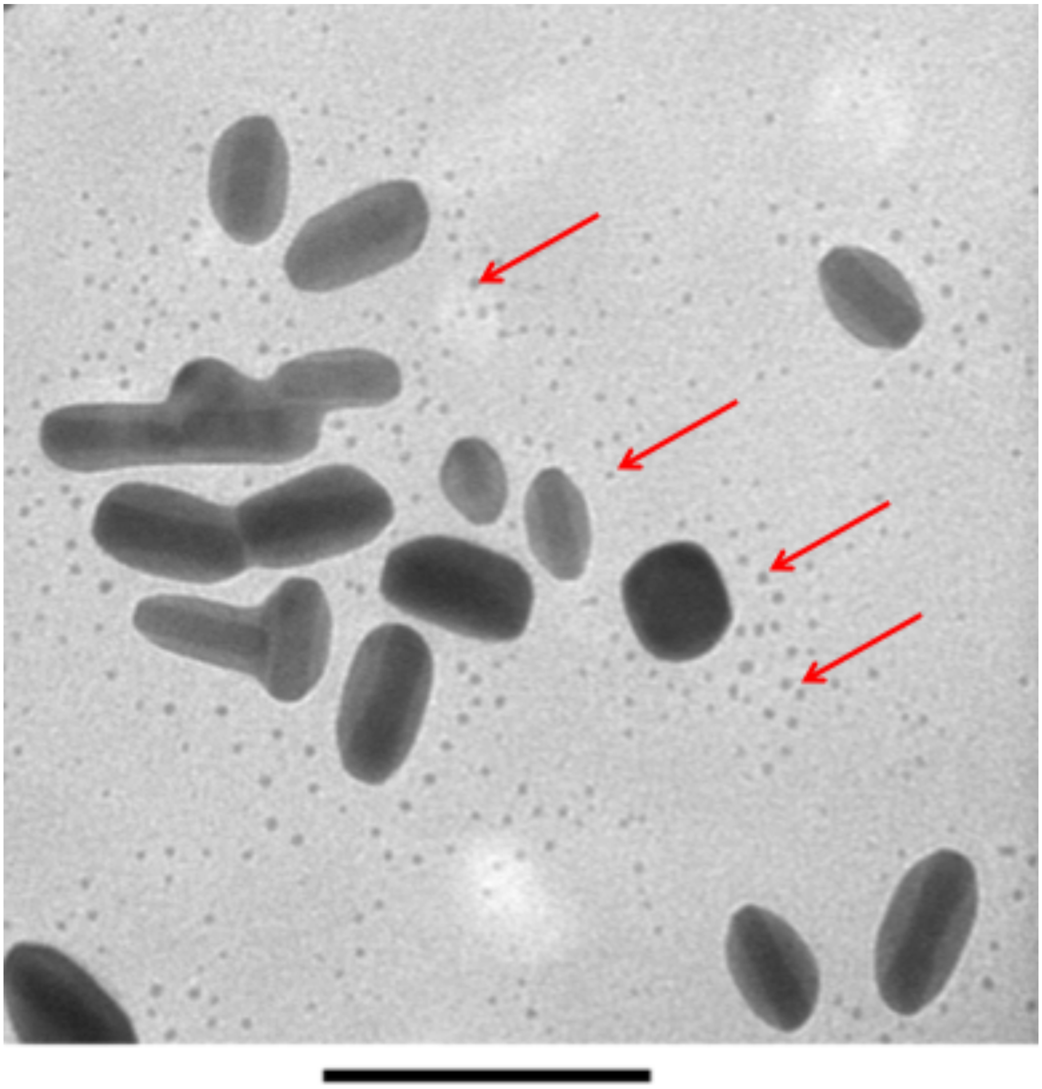

Supplementary Figure 6. Representative TEM image of thiol PEG2000 functionalized AuNR-Ag 585 nm incubated in serum at 37 °C leading to shell decomposition (red arrows). The scale bar is 100 nm.

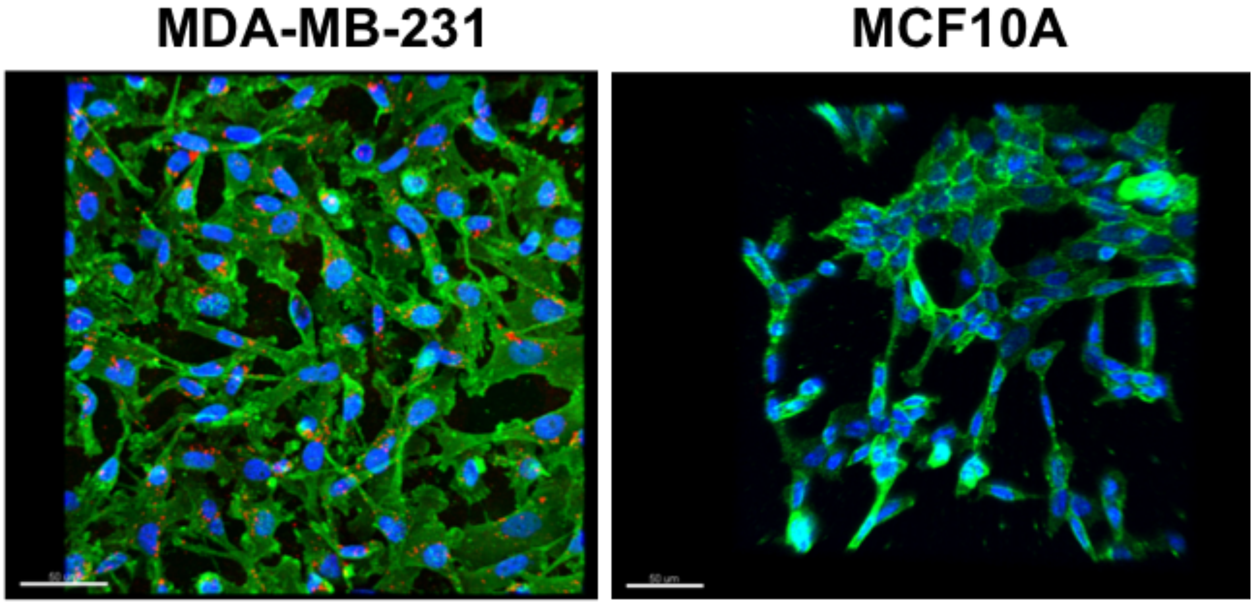

Supplementary Figure 7. Confocal fluorescence microscope images of MDA-MB-231(left) and MCF10A(right) cells incubated with 100 pM of OFRNPs for 16 hours. Cell nucleus and membrane was stained with Hoechst® 33342 (blue) and wheat germ agglutinin, AF488 conjugate (green) respectively. The uptake of OFRNPs(red) was higher in MDA-MB-231(left) compared to MCF10A(right). Scale bars are 50 μm.

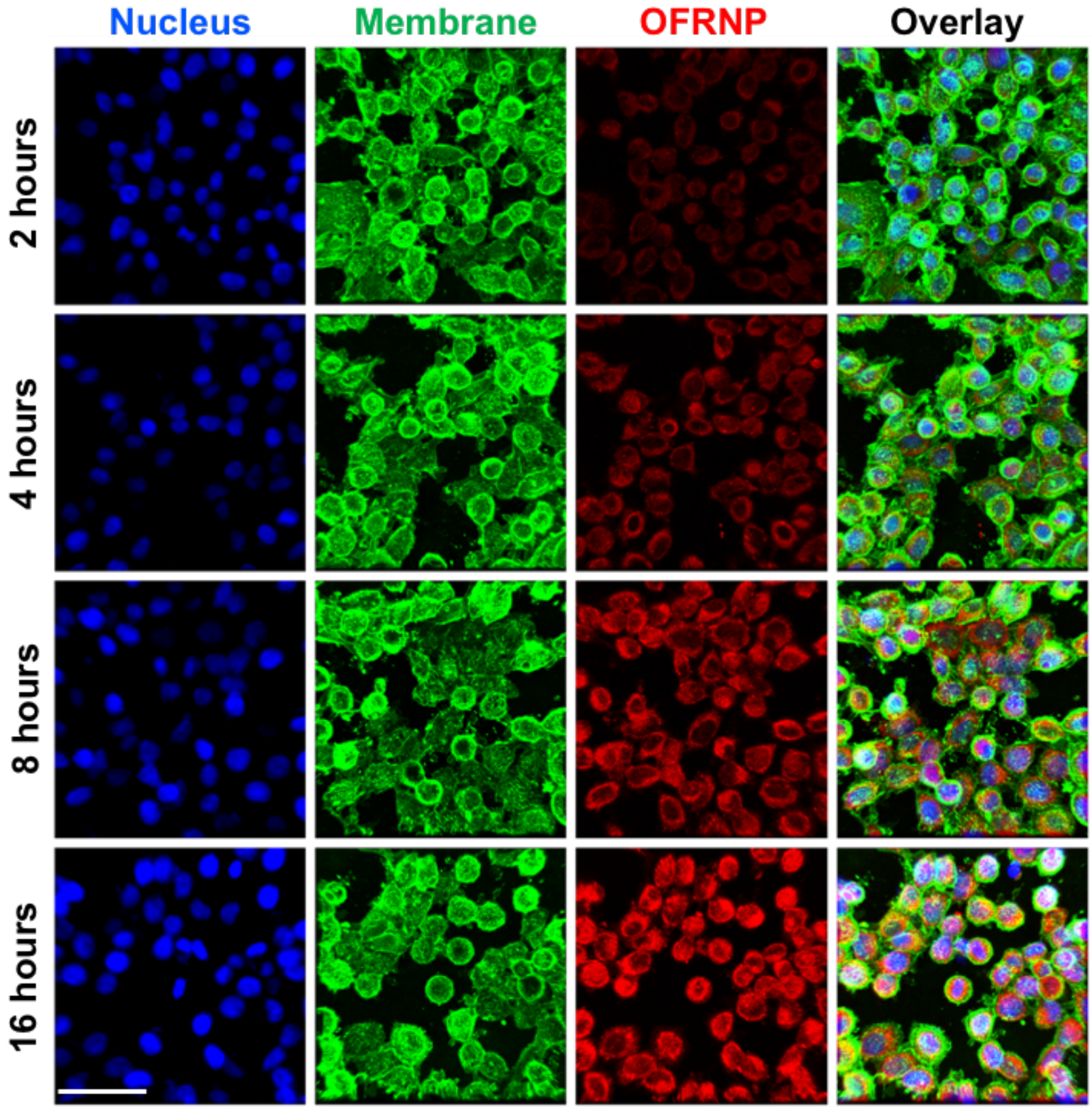

Supplementary Figure 8. Confocal fluorescence microscope images of MDA-MB-468 cells incubated with 100 pM of FRNPs at different time points at 2, 4, 8 and 16 hours. Longer incubation time increased the uptake of the OFRNPs (Red). Cell nucleus and membrane was stained with Hoechst® 33342 (blue) and wheat germ agglutinin, AF488 conjugate (green) respectively. The scale bar is 100 μm.

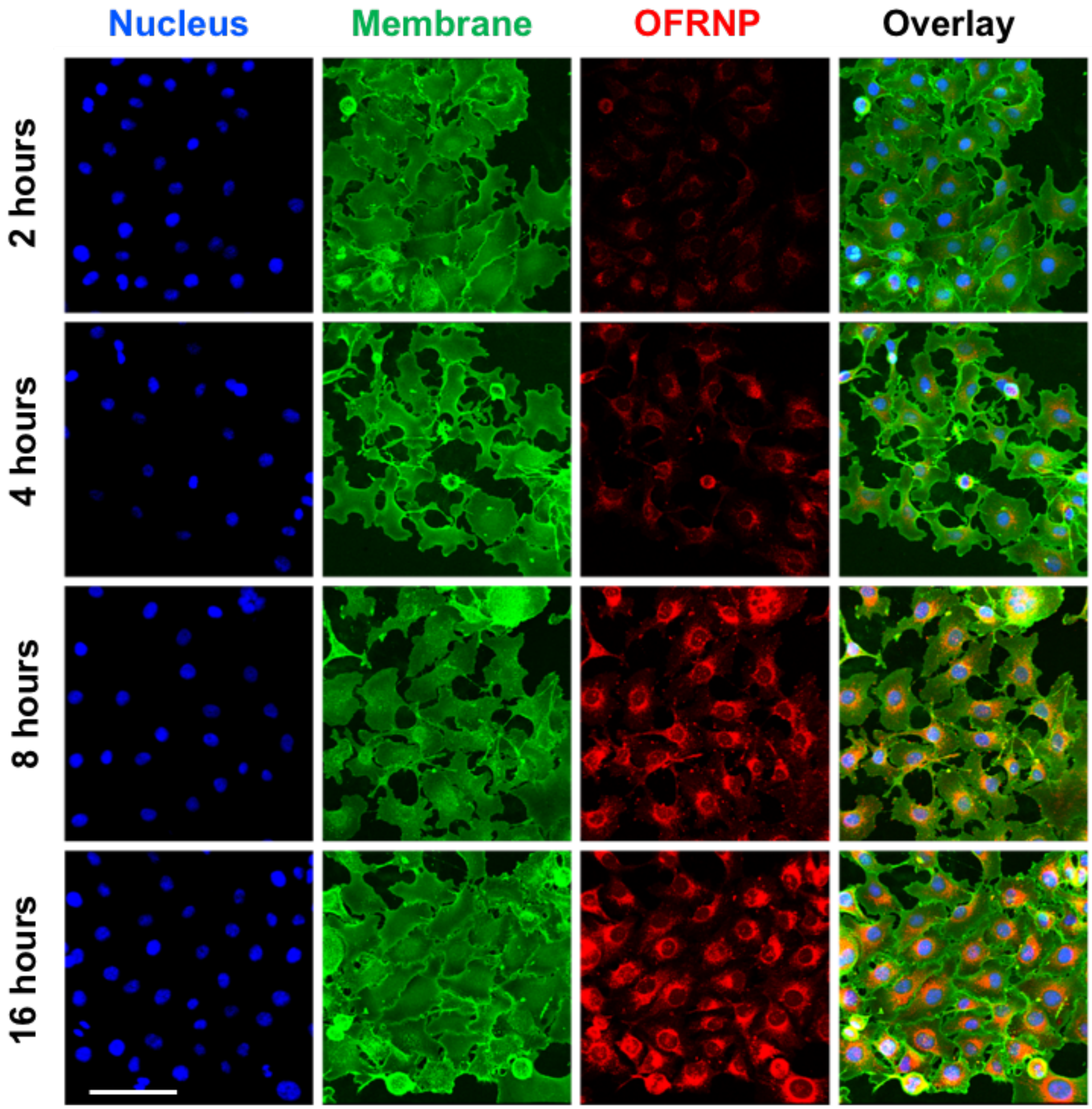

Supplementary Figure 9. Confocal fluorescence microscope images of SKOV-3 cells incubated with 100 pM of FRNPs at different time points at 2, 4, 8 and 16 hours. Longer incubation time increased the uptake of the OFRNPs (Red). Cell nucleus and membrane was stained with Hoechst® 33342 (blue) and wheat germ agglutinin, AF488 conjugate (green) respectively. The scale bar is 100 μm.

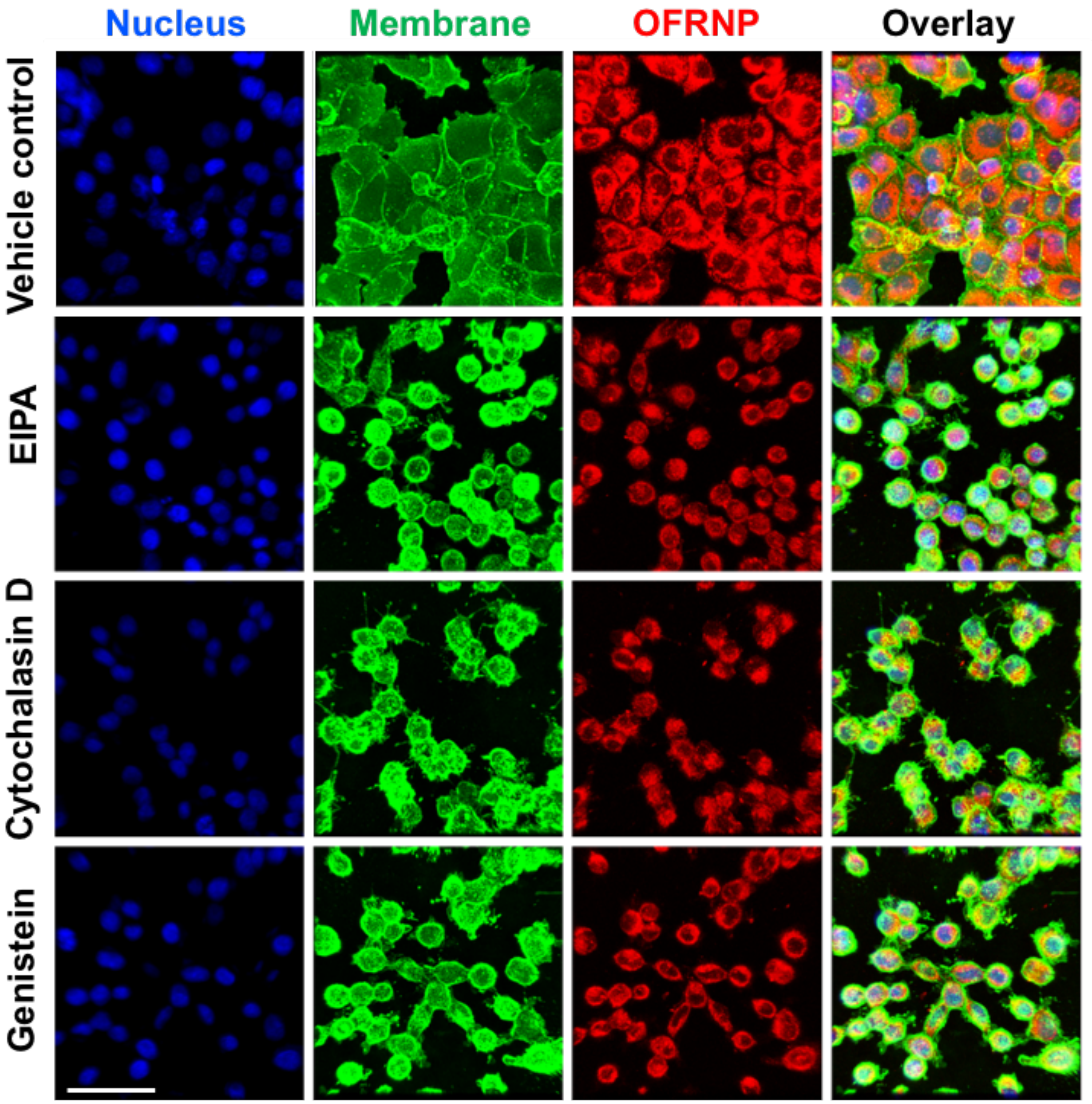

Supplementary Figure 10. Representative confocal fluorescence microscope images of MDA-MB-468 cells incubated with 100 pM of OFRNPs in the presence of different endocytosis inhibitors, 75 μM EIPA, 10 μg/ml Cytochalasien D, 2 μM Genistein. The scale bar is 100 μm.

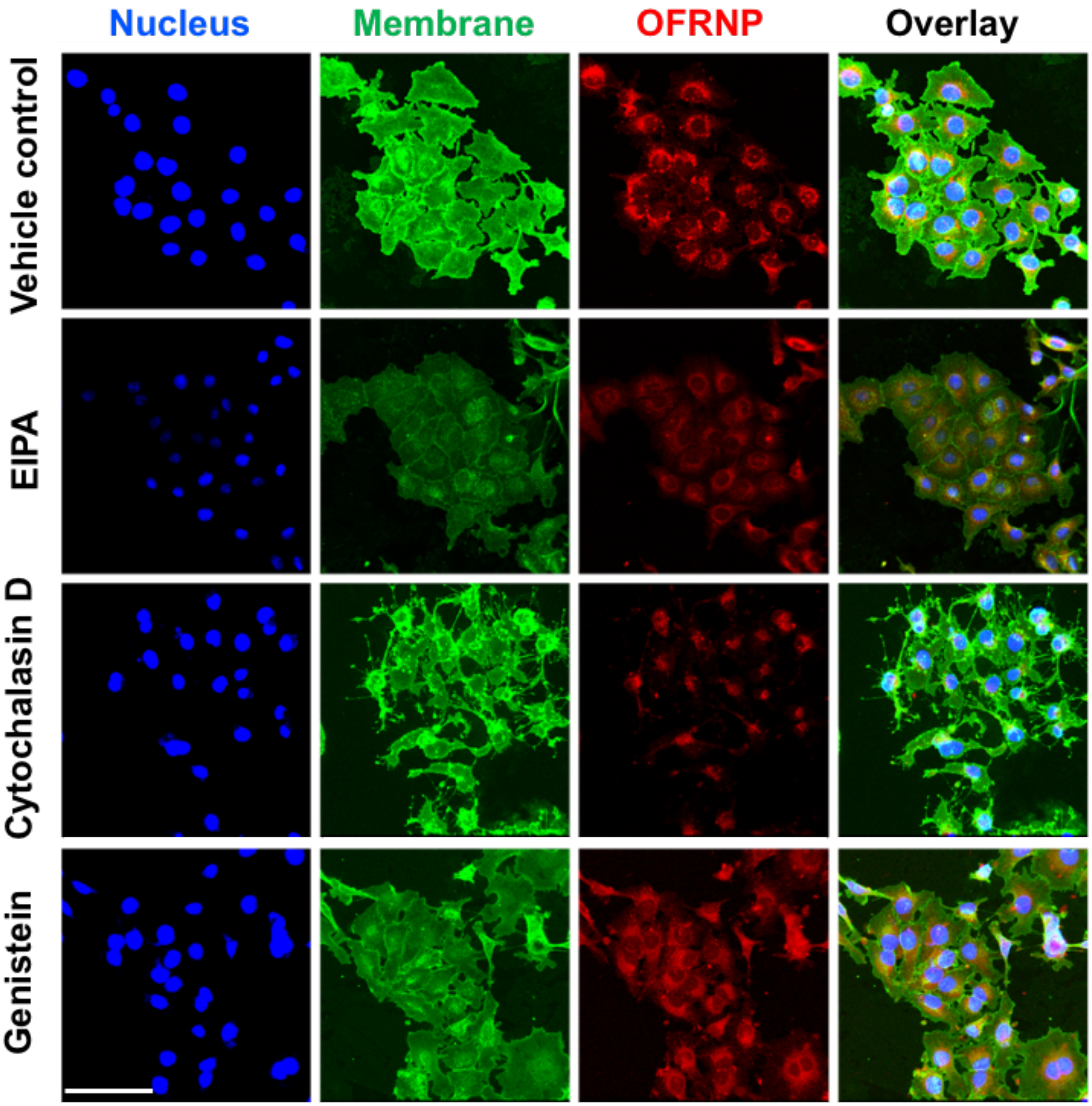

Supplementary Figure 11. Representative confocal fluorescence microscope images of SKOV-3 cells incubated with 100 pM of OFRNPs in the presence of different endocytosis inhibitors, 75 μM EIPA, 10 μg/ml Cytocalasien D, 2 μM Genistein. The scale bar is 100 μm.

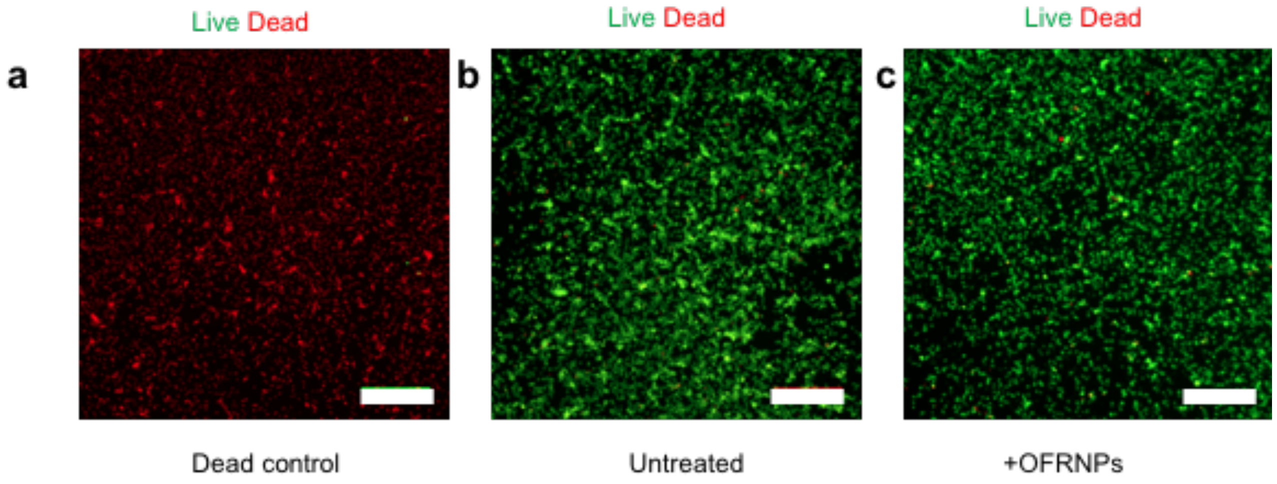

Supplementary Figure 12. Representative fluorescence microscope images of a Live-Dead assay of MDA-MB-231 cells (a) dead control (treated with 70% methanol) (b) untreated control (c) incubated with 500 pM of OFRNPs for 16 hours. These results exhibited no significant cytotoxicity of OFRNPs. The scale bars are 500 μm.

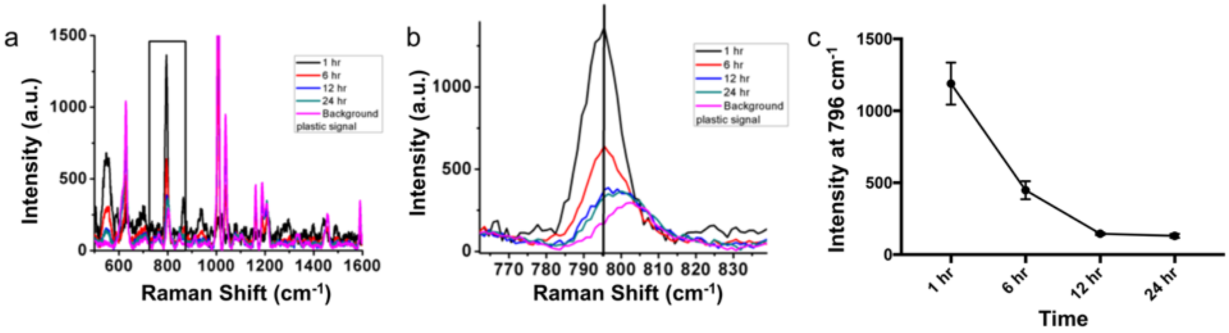

Supplementary Figure 13. Determination of blood circulation time: (a, b) Background subtracted Raman spectra of the blood of a mouse drawn at different time points post-injection of 200 μL of 10 nM OFRNPs (c) The intensities of 796 cm^{-1} (n=3) show the OFRNPs circulate in the blood up to 12 hours post injection.

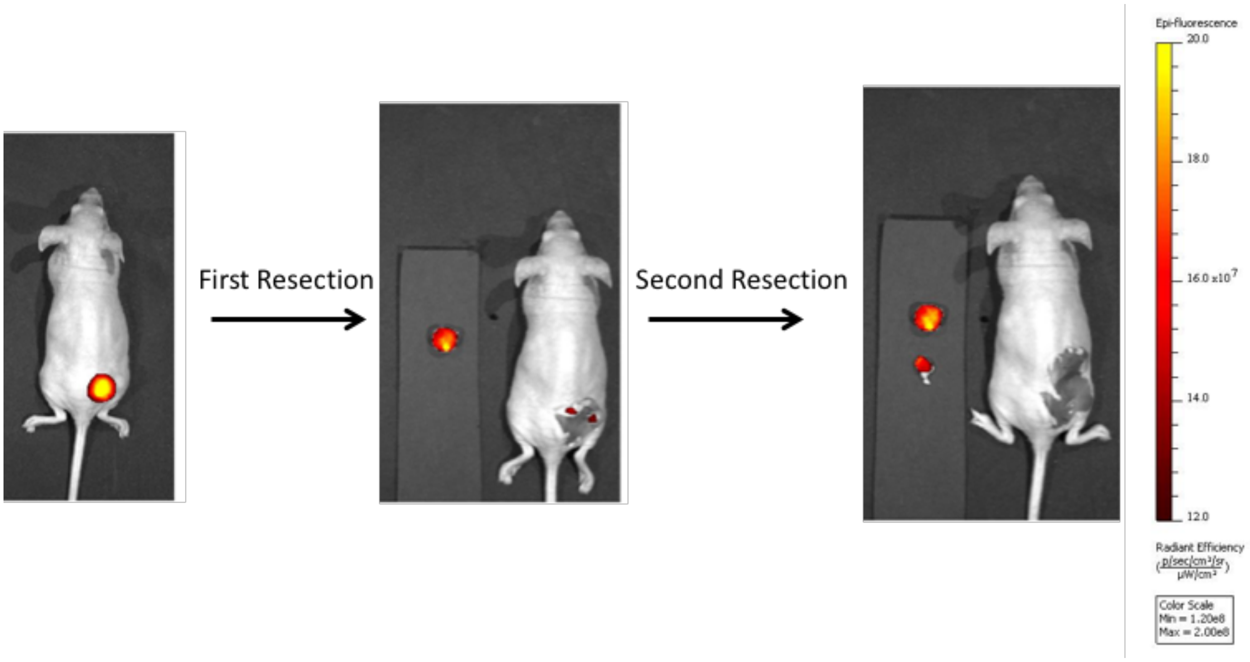

Supplementary Figure 14. Mock fluorescence guided surgery of ovarian cancer mouse models. NIR fluorescence images of mice 48 hours after intravenous administration of 200 μl (10 nM) OFRNPs. The visible contrast in tumor tissue guided surgical resection of tumor mass. There existed residual fluorescence positive tissue after the first resection. Therefore, a second resection was carried out to remove all the residual tumor tissue.

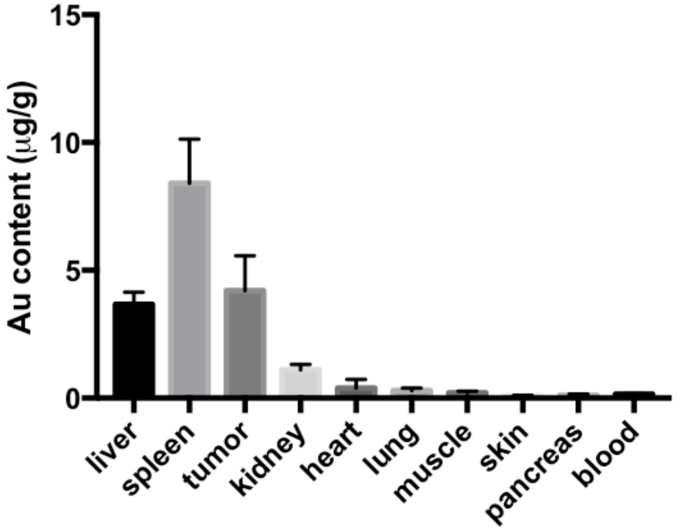

Supplementary Figure 15. Biodistribution of OFRNPs in ovarian cancer mice (n=3) measured by atomic absorption spectroscopy (AAS, Atomic Absorption Spectrometer Analyst 800). Liver and spleen have highest uptake while tumor tissue demonstrates selective localization of the NPs compared to normal tissue.

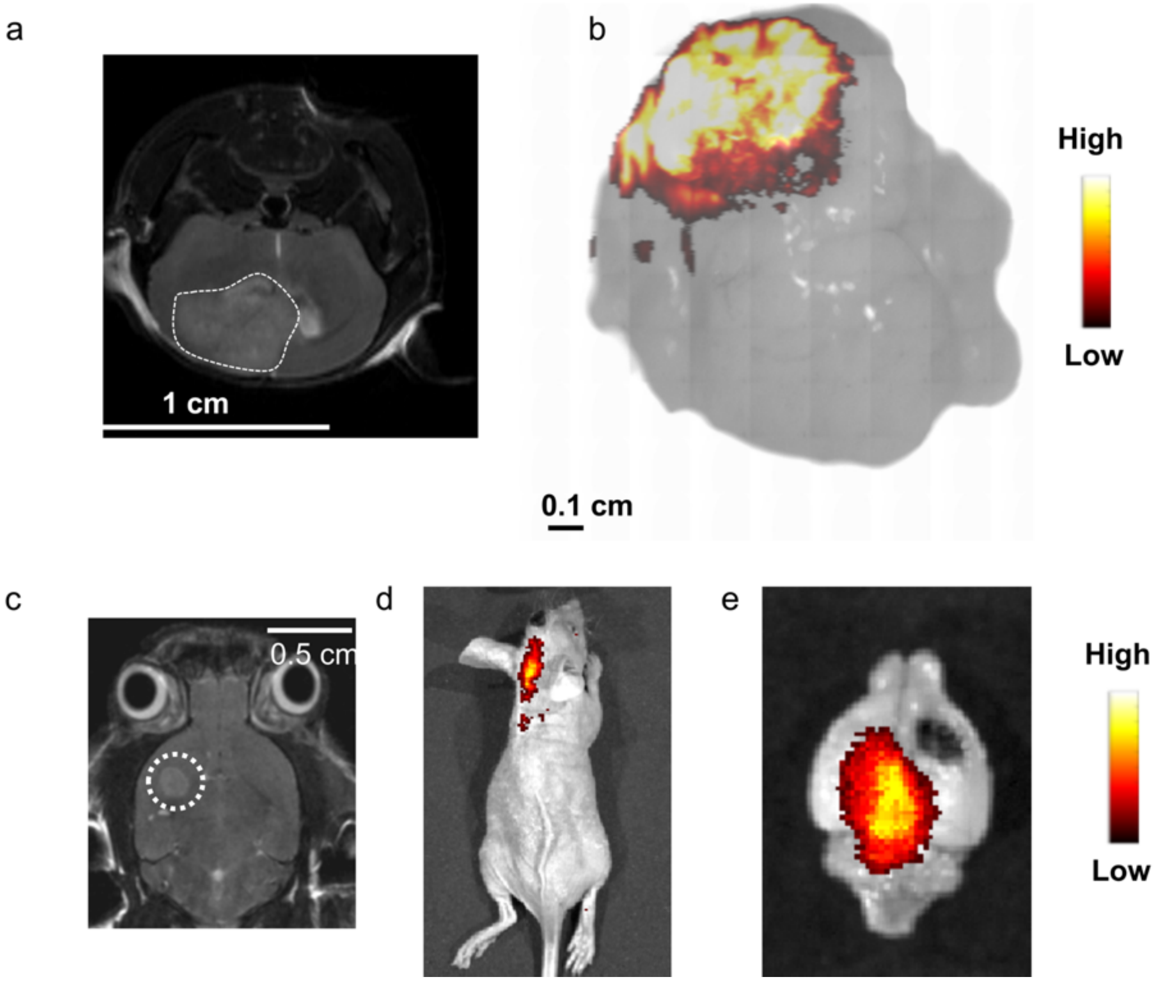

Supplementary Figure 16. (a) A typical coronal MRI image of a brain of tumor bearing RCAS/tv-a glioblastoma (GBM) mouse (Bruker Biospin Corp., Billerica, MA). (b) NIR fluorescence image of the ex vivo brain after intravenous administration of 200 μl (10 nM) OFRNPs showing selective accumulation of OFNPs in cancer tissue. (c) Dorsal MRI image of a brain of tumor bearing syngeneic immunocompetent GBM mouse model (CT-2A cell line inoculated). (d) NIR fluorescence image of whole mouse after 16 hours post injection shows fluorescence signal localized in the brain. (e) Fluorescence image of Ex-vivo brain further shows localized fluorescence signal from the brain.

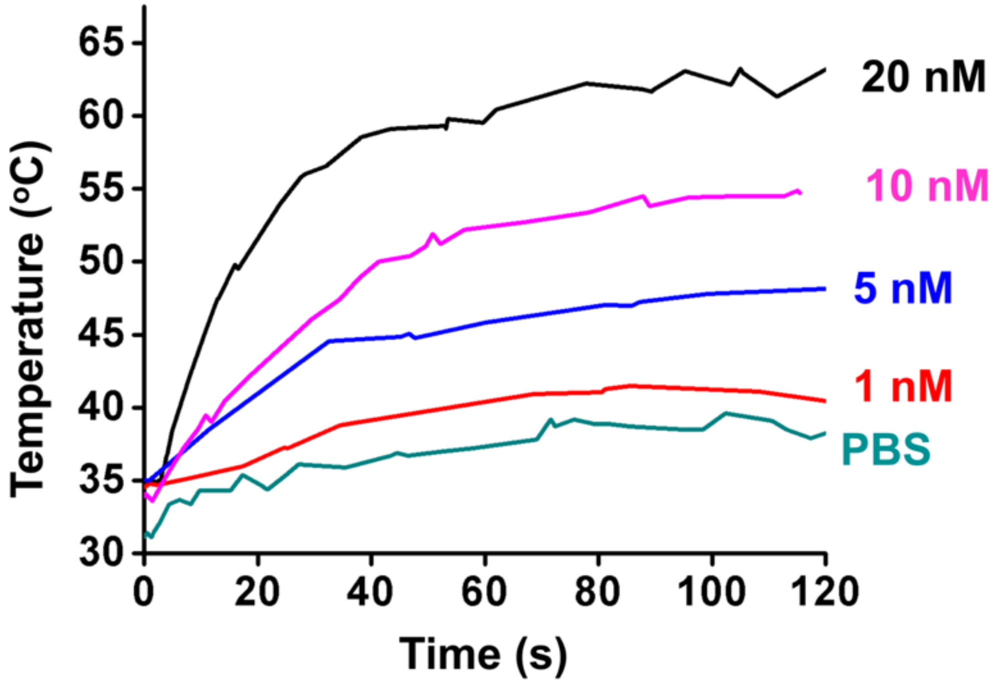

Supplementary Figure 17. Average temperature of the tumor area during photothermal therapy of mice injected with different concentrations (200 μL) of OFRNP compared to the PBS injected mouse. Temperature increase is higher for mice injected with higher concentration of OFRNPs.

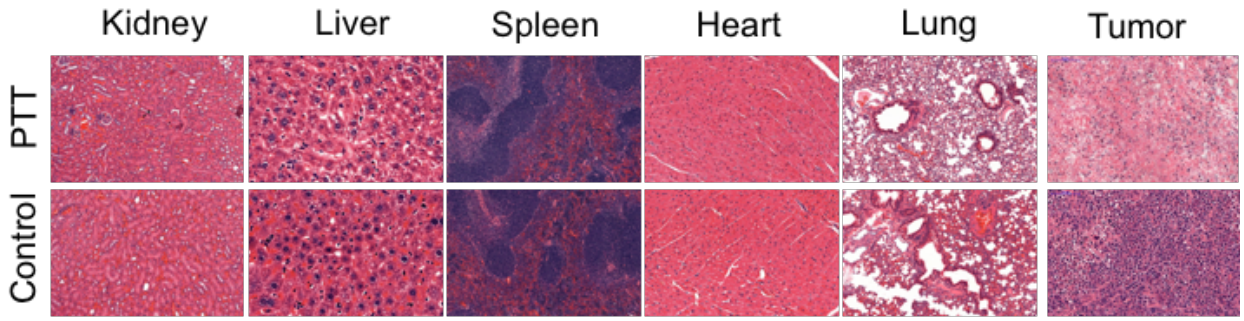

Supplementary Figure 18. Histopathological evaluation using H&E staining. Major organs were harvested 2 weeks post i.v. injection of OFRNPs and PTT. Control mice were injected with PBS. No major differences in organs were found in treatment and control group except the tumor was found to be necrotic in PTT group.

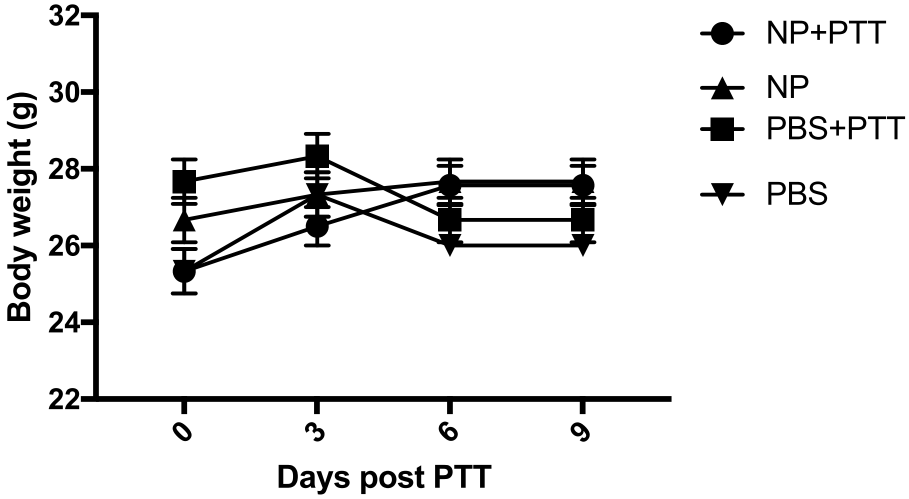

Supplementary Figure 19. Body weight of four different treatment groups exhibited no significant changes (n=3).

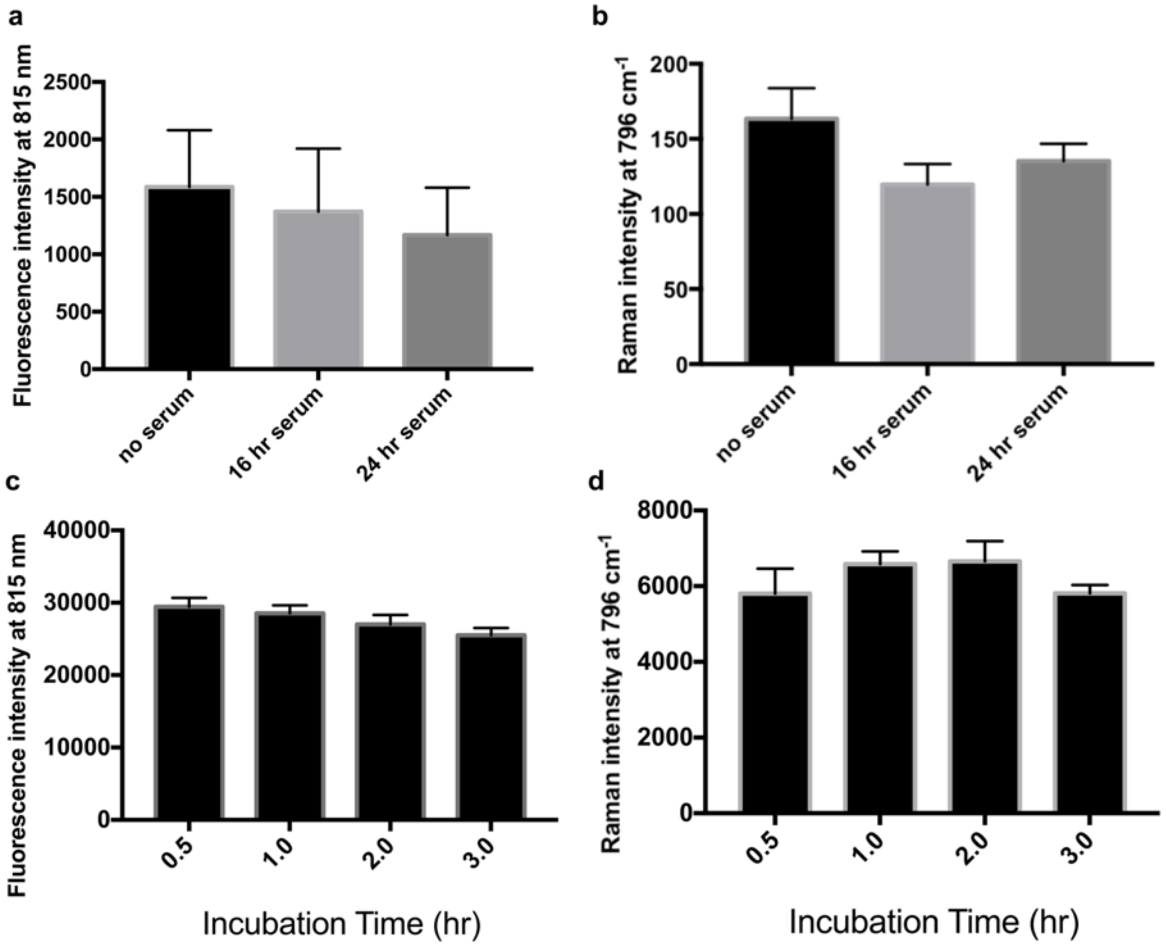

Supplementary Figure 20. Serum stability of OFRNPs. (a) The fluorescence intensity at 815 nm decreased by 27% over 24 hrs (b) the background subtracted Raman intensity at 796 cm^{-1} dropped by ~26% over 24 hrs. (c, d) Short term serum stability show only ~13% decrease in fluorescence intensity and no significant change in Raman intensity. All data represent triplicate of the measurements.

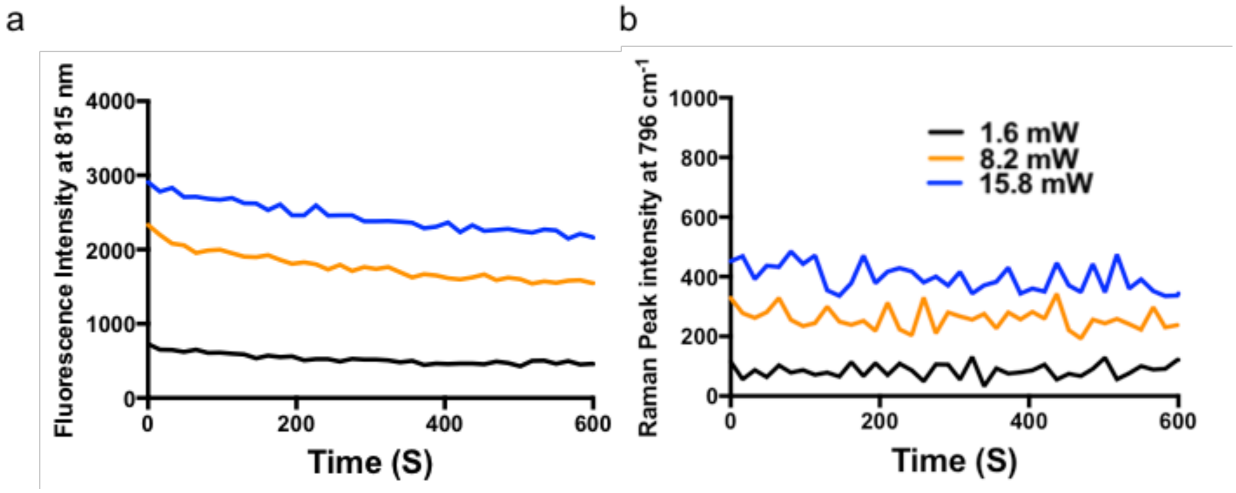

Supplementary Figure 21. Photo stability of 1nM of OFRNPs in tissue phantom under different excitation laser power (1.6 mW, 8.2 mW and 15.8 mW) (a) Fluorescence intensity at 815 nm and (b) Raman peak intensity at 796 cm^{-1} profile with time.

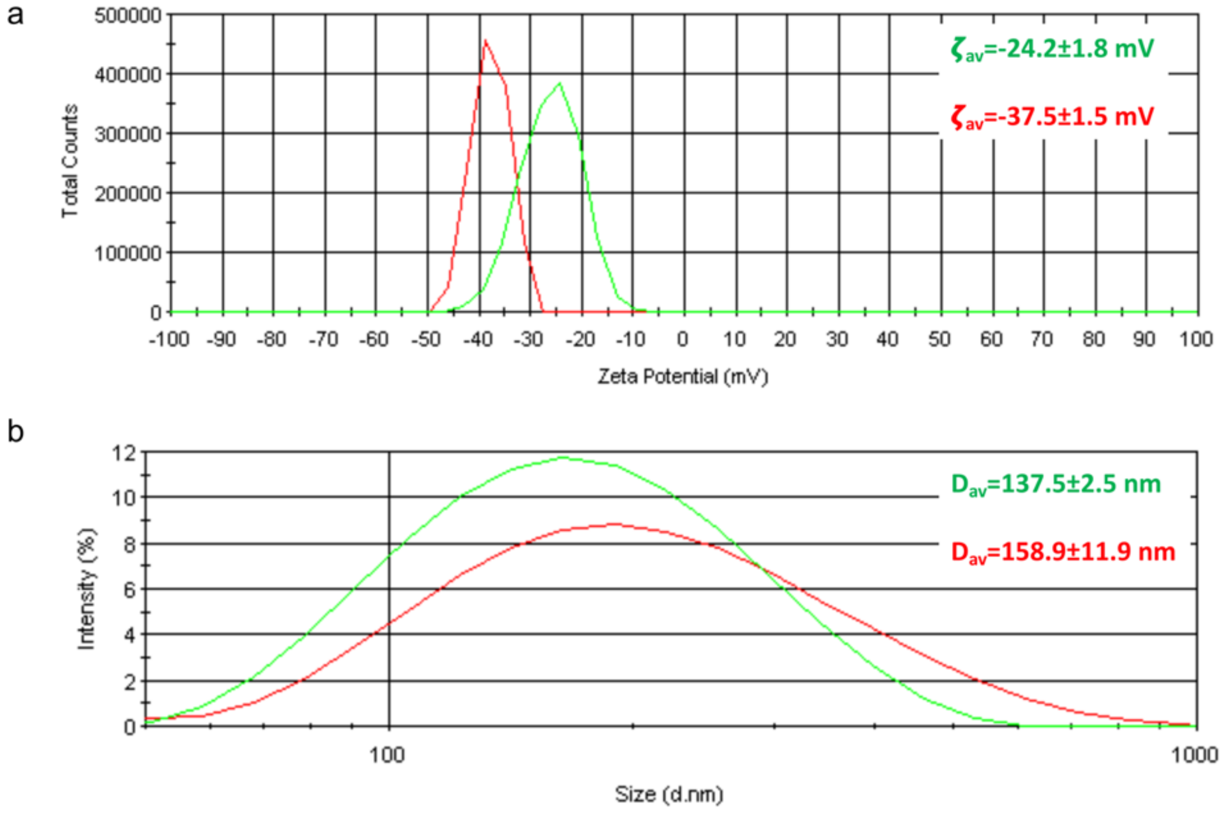

Supplementary Figure 22. (a) Plot of zeta potential of OFRNPs (green) and OFRNPs incubated in serum for 24 hours at 37 °C (red). (b) Plot of average hydrodynamic diameter of OFRNPs (green) and OFRNPs incubated in serum for 24 hours at 37 °C (red).

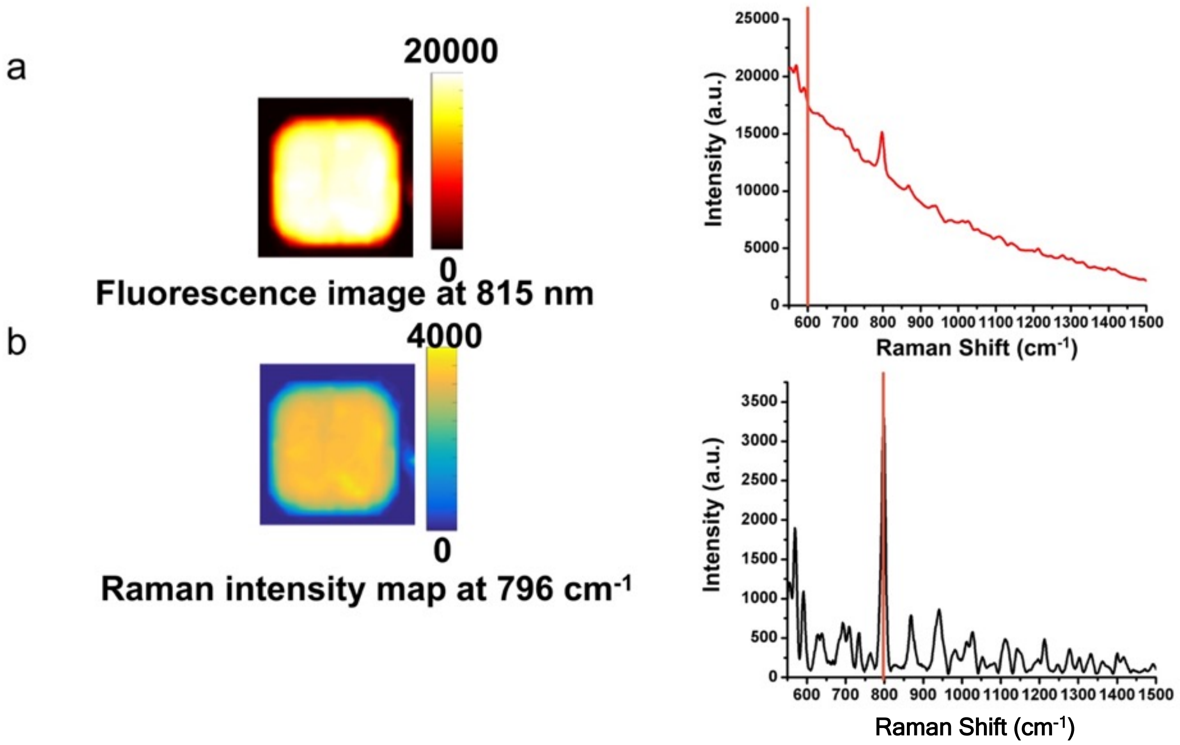

Supplementary Figure 23. Dual-mode imaging of 1 pM of OFRNPs in tissue phantom using a Raman microscope. We imaged the phantom using the same setup for the ex vivo imaging in InVia Raman microscope (Renishaw, 161 mW laser power, with 1.5 s acquisition time, using the Stream Line high-speed acquisition mode). (a) After the point-by-point data acquisition, we processed the data using graphical user interface developed in-house to plot the total intensity at Raman shift 600 cm^{-1} (815 nm). This image corresponds to a fluorescence image. (b) The point-by-point spectra were background subtracted using Whittaker filter (with width λ = 200 cm^{-1}) to remove the broad fluorescence background signal and produce purely Raman spectra. We plotted the intensity at 796 cm^{-1}.

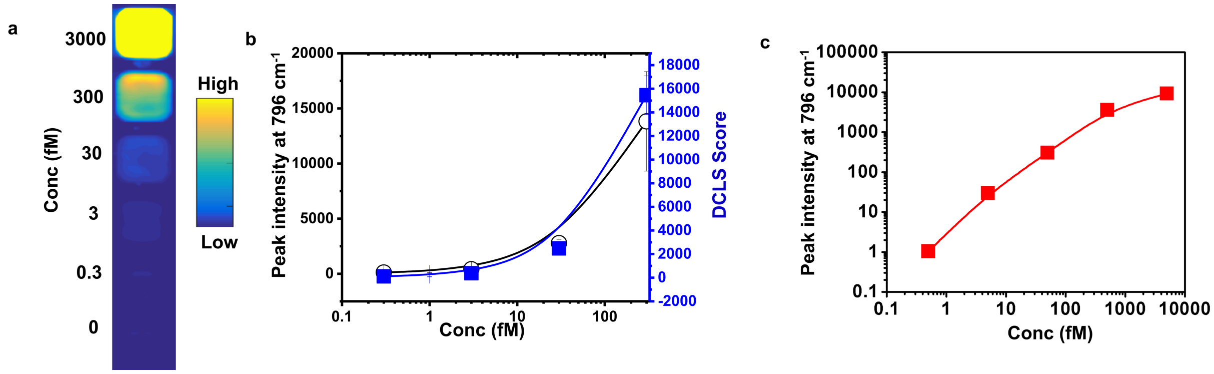

Supplementary Figure 24. (a) DCLS Raman map of a tissue phantom with different concentrations of FRNPs acquired using a phantom using an InVia Raman microscope (Renishaw, 161 mW laser power, with 1.5 s acquisition time, using the Stream Line high-speed acquisition mode). DCLS identifies the presence of a particular spectral signature. In order to generate the DCLS model, the reference spectra were preprocessed by baseline subtraction using a Whittaker filter (with width λ = 200 cm^{-1}) and subjected to L1-norm (normalization by the area), followed by a Savitzky–Golay derivative filter (second-degree polynomial fit, first-order derivative, width = 15 steps). (b) The limit of detection of OFRNPs in the tissue phantom was determined to be between 3-10 fM. (c) The limit of detection of OFRNPs in blood was determined to be ~5 fM.

Supplementary References

1. Nikoobakht B, El-Sayed MA. Preparation and Growth Mechanism of Gold Nanorods (NRs) Using Seed-Mediated Growth Method. *Chemistry of Materials* **15**, 1957-1962 (2003).

2. Cleveland CL, Landman U, Shafigullin MN, Stephens PW, Whetten RL. Structural evolution of larger gold clusters. *Z Phys D: At Mol Clusters* **40**, 503-508 (1997).

3. Luedtke WD, Landman U. Structure, Dynamics, and Thermodynamics of Passivated Gold Nanocrystallites and Their Assemblies. *J Phys Chem* **100**, 13323-13329 (1996).

4. Nelson HC, Finch JT, Luisi BF, Klug A. The structure of an oligo(dA).oligo(dT) tract and its biological implications. *Nature* **330**, 221-226 (1987).

5. Wang J, Cieplak P, Kollman PA. How well does a restrained electrostatic potential (RESP) model perform in calculating conformational energies of organic and biological molecules? *J Comput Chem* **21**, 1049 (2000).

6. Pérez A*, et al.* Refinement of the AMBER force field for nucleic acids: improving the description of alpha/gamma conformers. *Biophys J* **92**, 3817-3829 (2007).

7. Hautman J, Klein ML. Simulation of a monolayer of alkyl thiol chains. *J Chem Phys* **91**, 4994-5001 (1989).

8. Graen T, Hoefling M, Grubmüller H. AMBER-DYES: Characterization of Charge Fluctuations and Force Field Parameterization of Fluorescent Dyes for Molecular Dynamics Simulations. *J Chem Theory Comput* **10**, 5505-5512 (2014).

9. Agrawal PM, Rice BM, Thompson DL. Predicting trends in rate parameters for self-diffusion on FCC metal surfaces. *Surf Sci* **515**, 21-35 (2002).

10. Schaftenaar G, Noordik JH. *J Comput Aided Mol Des* **14**, 123-134 (2000).

11. Abraham MJ*, et al.* GROMACS: High performance molecular simulations through multi-level parallelism from laptops to supercomputers. *SoftwareX* **1-2**, 19-25 (2015).

12. Sagui C, Darden TA. MOLECULAR DYNAMICS SIMULATIONS OF BIOMOLECULES: Long-Range Electrostatic Effects. *Annu Rev Biophys Biomol Struct* **28**, 155-179 (1999).

13. Bussi G, Donadio D, Parrinello M. Canonical sampling through velocity rescaling. *J Chem Phys* **126**, 014101 (2007).

14. Hess B, Bekker H, Berendsen HJC, E. JG. LINCS: A linear constraint solver for

molecular simulations. *J Comput Chem* **18**, 1463-1472 (1997).

15. Miyamoto S, Kollman PA. Settle: An analytical version of the SHAKE and RATTLE algorithm for rigid water models. *J Comput Chem* **13**, 952-962 (1992).

16. Drew HR*, et al.* Structure of a B-DNA dodecamer: conformation and dynamics. *Proc Natl Acad Sci U S A* **78**, 2179-2183 (1981).

17. Humphrey W, Dalke A, Schulten K. VMD: Visual molecular dynamics. *Journal of Molecular Graphics* **14**, 33-38 (1996).
