# Supplementary material for: DNA-enabled rational design of fluorescence-Raman bimodal nanoprobes for cancer imaging and therapy
Source: Nat Commun. 2019 Apr 26;10:1926. doi: 10.1038/s41467-019-09173-2 (PMC6486596; doi:10.1038/s41467-019-09173-2)
Supplement: Supplementary file 2 — Description of Additional Supplementary Files [file 41467_2019_9173_MOESM2_ESM.docx]

**Description of Supplementary Files**

**File Name:** Supplementary Movie 1

**Description:** Time lapses of molecular dynamics simulation of HS-(PS)A6-Cy7 on 1.2 nm AuNP.

**File Name:** Supplementary Movie 2

**Description:** Time lapses of molecular dynamics simulation of HS-(PO)A6-Cy7 on 1.2 nm AuNP.

**File Name:** Supplementary Movie 3

**Description:** Time lapses of molecular dynamics simulation of HS-(PO)TCGCGC-Cy7 on 1.2 nm AuNP.
